# Differential Diagnosis of Anemia Using the Erythrocyte Filterability Method

**DOI:** 10.3390/ijms27177654

**Published:** 2026-08-26

**Authors:** Elena I. Sinauridze, Elizaveta A. Bovt, Dmitry S. Prudinnik, Ivan A. Dolgikh, Larisa Koleva, Soslan S. Shakhidzhanov, Nikita S. Kushnir, Anna S. Suvorova, Evgeniy S. Protasov, Evgeniya A. Brovkina, Ivan A. Chabin, Svetlana G. Kapranova, Svetlana A. Plyasunova, Nataliya S. Smetanina, Fazoil I. Ataullakhanov

**Affiliations:** 1 Dmitriy Rogachev National Medical Research Center of Pediatric Hematology, Oncology, and Immunology, Ministry of Healthcare, Samora Machel Street, 1, GSP-7, Moscow 117198, Russia; maddima285@gmail.com (D.S.P.); andrei4rus@mail.ru (I.A.D.); larulea@mail.ru (L.K.); shakhidzhanov.s@yandex.ru (S.S.S.); x-nekit@yandex.ru (N.S.K.); makagonova.anna@mail.ru (A.S.S.); protasov_evgenii@mail.ru (E.S.P.); evgenia.brovkina@dgoi.ru (E.A.B.); i.a.chabin@yandex.ru (I.A.C.); svetlana.kapranova@dgoi.ru (S.G.K.); svetlana.plyasunova@dgoi.ru (S.A.P.); nataliya.smetanina@dgoi.ru (N.S.S.); 2 Center for Theoretical Problems of Physicochemical Pharmacology, Russian Academy of Sciences, Srednyaya Kalitnikovskaya Street, 30, Moscow 109029, Russia

**Keywords:** red blood cell, hereditary hemolytic anemia, differential diagnosis, hereditary spherocytosis, hereditary stomatocytosis, pyruvate kinase deficiency, filterability of erythrocytes, EMA test, osmotic resistance of erythrocytes, sphericity index

## Abstract

Hereditary hemolytic anemias (HHAs) have very similar clinical manifestations, complicating differential diagnosis and determining treatment strategies, such as splenectomy. To differentiate between hereditary spherocytosis (HS), pyruvate kinase deficiency (PKD), and hereditary stomatocytosis (HSt), we proposed measuring erythrocyte filterability using membrane filters with a pore diameter of 3 or 3.5 μm. Our modified method provides improved reproducibility, reducing the scattering in results caused by differences in the filters used. Erythrocytes’ filterability differs significantly between patients with HS, HSt, and PKD (medians and 95% confidence intervals were 0.025 [0.004; 0.100] (*n* = 86); 0.645 [0.520; 0.730] (*n* = 10), and 0.760 [0.700; 0.782] (*n* = 26), respectively). The specificity of the filterability test for the HS diagnosis was 99.1%, comparable with the best available tests, while its sensitivity (85.4%) was slightly lower than that of some alternative methods. Only the filterability test was able to identify a second subgroup of HS patients (14.0%) with higher filterability (close to normal), but the diagnosis of HS in these patients could be confirmed by reduced filterability on a 3-μm filter. This subgroup requires further detailed study. The proposed method for measuring filterability is promising for diagnosing HS. It has high sensitivity and specificity and can be performed quickly without expensive equipment.

## 1. Introduction

Anemia is a condition that reduces the oxygen-carrying capacity of the blood due to a decrease in hemoglobin concentration and/or a decrease in the number of red blood cells (erythrocytes, RBCs), which, in turn, can cause hypoxia and hypoxemia. One of the main mechanisms of anemia—hemolytic anemia (HA)—is associated with accelerated destruction and, consequently, a shortened lifespan of RBCs in the bloodstream.

Hereditary hemolytic anemias (HHAs) can have various causes related to abnormalities in erythrocyte enzymes, membranes, or hemoglobin structure [1,2,3]. These causes vary, but the clinical signs of different hemolytic anemias are often very similar. Typically, these include hemolysis of RBCs, a decrease in their number, a decrease in the concentration of hemoglobin in the blood (to <110 g/L of blood), jaundice, an increase in the number of reticulocytes, splenomegaly, the development of cholelithiasis, an increase in the concentration of unconjugated bilirubin in the serum, and decreased levels of haptoglobin, lactate, and pyruvate [4].

To perform their physiological function (transport of gases), erythrocytes must circulate in the bloodstream for a long time, reaching all tissues, including repeatedly passing through narrow capillaries and splenic sinuses, the diameter of which is two to three times smaller than the diameter of erythrocytes. This is ensured by the unique flexibility and deformability of RBCs, which is a function of the structure of cytoskeletal proteins and depends on maintaining erythrocyte volume and the ratio of membrane surface area to cell volume. Inability to deform leads to a shortened RBC lifespan and, consequently, to hemolytic anemia [5,6].

First of all, erythrocyte deformability affects blood microrheology rather than macrorheology, since in narrow capillaries, the forces acting on erythrocytes (shear stress) can be 10–100 times greater than those acting on them in large vessels [7]. Currently, various methods are used to determine erythrocyte deformability. A detailed description of these methods can be found in reviews [5,8,9,10,11]. However almost all these methods, are not suitable for routine diagnostics of erythrocytes’ capability to pass through narrow capillaries.

Some methods measure the deformation of erythrocytes (their deviation from the discoid shape) when a cell suspension flows in a high-density medium through a channel whose diameter is greater than the diameter of the erythrocyte. This approach allows us to determine only the average parameters of deformability in the entire population of RBCs, but is unable to identify a small proportion of cells (~0.1–1% of their total number) with reduced deformability, although these cells can seriously affect the microrheology of blood in the body. Such methods include ektacytometry (in different versions) [12,13,14,15] or real-time deformability cytometry [16], when images of RBCs flowing through a flowcytometer chamber taken under a microscope with a very high-speed camera are analyzed.

Other methods (erythrocyte aspiration into a microcapillary [17], atomic force microscopy [18,19], optical tweezers [20,21,22], etc.) are not suitable for routine diagnostics, because they work with single cells, and, therefore, in order to reliably identify a small proportion of poorly deformable cells using such methods, it is necessary to process 50–70 thousand individual cells, which is very time-consuming and labor-intensive. Thus, to improve the diagnosis of the hemolytic anemia causes, it is necessary to have a method that allows us to determine the ability of RBCs to deform and pass through narrow capillaries, since it is this ability that determines the microrheology of the blood.

Recently developed microfluidics methods [23,24,25,26] measure the flow rate of erythrocyte suspensions not through filters, but through microfluidic chips with channels of varying thickness and geometry, simulating the structure of capillaries and interendothelial spaces in the spleen (under controlled pressure). The deformation process is recorded using high-speed video microscopy. These methods claim to measure the flow of erythrocyte suspensions under conditions as close as possible to those actually occurring in vivo. However, their application is severely limited by the need for specialized, expensive equipment, the complexity of manufacturing and the high cost of microfluidic chips, and, most importantly, the difficulty of standardizing experimental conditions, which hinders the comparison of results obtained both within and between laboratories.

The most suitable method for characterizing the ability of RBCs to pass through narrow capillaries is measuring the filterability of RBCs through artificial membrane filters with a pore diameter of 3 to 5 μm, as it directly measures this ability. However, this method has never been used to diagnose hereditary hemolytic anemias, as its versions described in the literature have low reproducibility. This is because traditional methods for assessing filterability only measure the time required for a sample (blood or RBCs suspension) to pass through the filter. The experiment is repeated each time on a new filter. If whole blood is used in the experiment, the filter pores become clogged with larger cells (leukocytes) or cell aggregates (produced primarily from platelets). If washed RBCs are used, the time required for the cell suspension to pass through the filter depends heavily on the quality of the filter (primarily on the diameter and density of the pores). However, this quality varies significantly even within a single set of filters. All this leads to significant non-reproducibility of results even within a single laboratory [27,28,29]. Thus, the method for measuring erythrocyte filterability needs significant improvement.

This study, for the first time, proposes using a modified method for measuring RBC filterability through artificial membrane filters with a pore diameter of 3.5 and 3.0 μm for the differential diagnosis of hereditary spherocytosis (HS), hereditary stomatocytosis (HSt) and pyruvate kinase deficiency (PKD). The sensitivity of this method was increased by using a washed red blood cell suspension instead of whole blood, as well as by reducing the variability of results due to the imperfections of the filters used. For this purpose, we determined the filterability of each suspension as the ratio of the time required for a buffer solution to pass through the filter to the time required for an equal volume of RBC suspension to pass through the same filter. This made it possible to automatically take into account the heterogeneity of the filters used.

**Figure 1:**
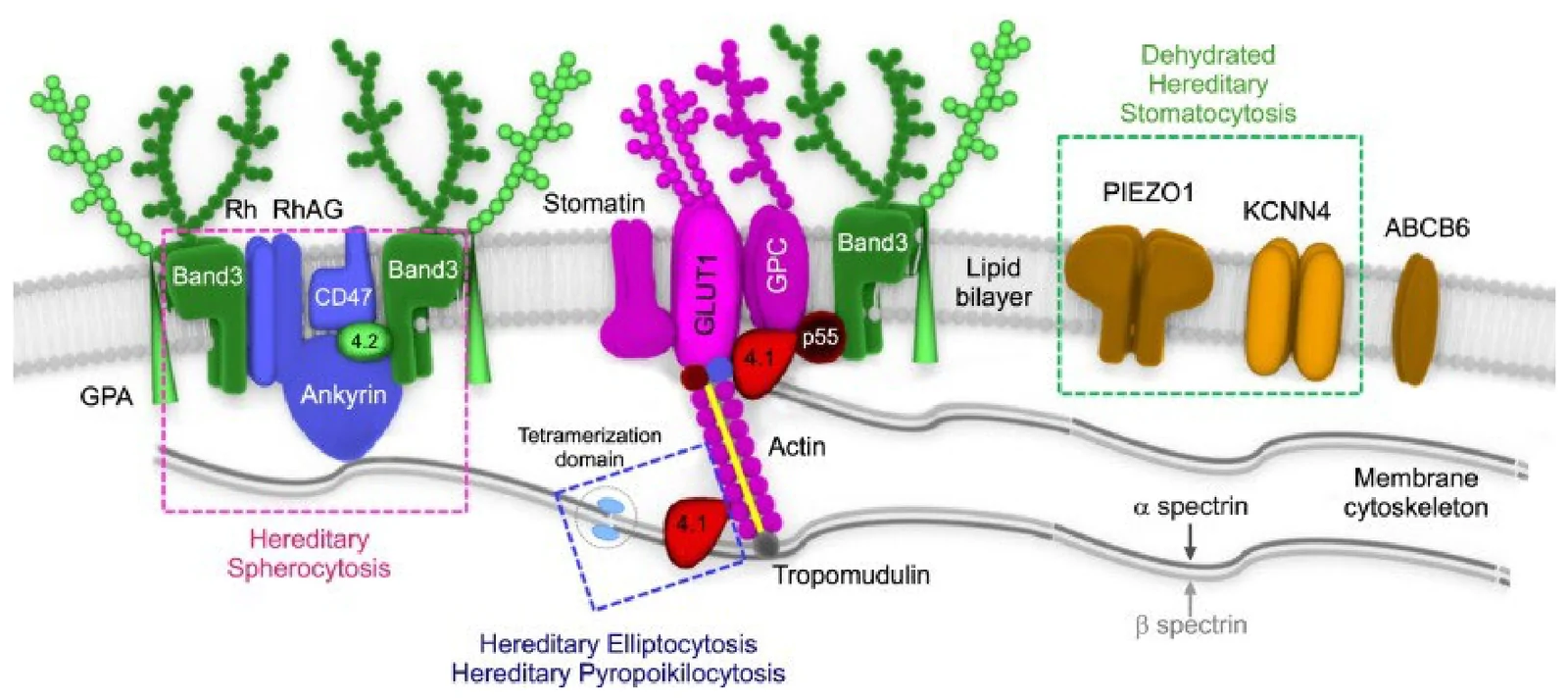
Schematic representation of proteins (separate ion channel proteins (PIEZO1 and KCNN4) or complexes of erythrocyte membrane cytoskeleton proteins), that are responsible for the development of various erythrocyte membranopathies. The abbreviations used: GPA, GPC—glycophorin A and C, respectively; Band 3—band 3 protein, Rh—rhesus factor protein, RhAG—Rh factor-associated glycoprotein; 4.1—protein 4.1R; GLUT1—glucose transporter; 4.2—protein 4.2; CD47—cellular determinants 47; p.55—protein 55; PIEZO1 is a mechanosensitive channel that forms a pore in the membrane through which ions, ATP, other small molecules, and water can be transported; KCNN4 is a Ca^2+^-activated potassium channel (Gardos channel). The dotted frames indicate protein complexes, disturbances in which cause certain types of diseases. Reproduced from [33] under CC BY-NC 4.0 license.

The mechanisms underlying anemia in the pathologies studied here differ. While erythrocyte metabolism and energy balance are disrupted in PKD due to decreased activity of the glycolytic enzyme pyruvate kinase, anemias in HS and HSt are caused by genetic mutations that cause a qualitative or quantitative deficiency of certain membrane proteins (Figure 1). In HS, interactions between proteins that bind the cytoskeleton of the erythrocyte inner membrane and its outer lipid bilayer (proteins of complexes that determine vertical interactions in the membrane) are disrupted [1]. Stomatocytoses are disorders of erythrocyte hydration. They constitute a heterogeneous group of disorders associated with disturbances in the water–salt balance of erythrocytes due to changes in membrane permeability for potassium and sodium ions, leading to changes in erythrocyte volume [30,31,32].

Any membrane abnormalities are accompanied by a decrease in the erythrocytes ability to deform while passing through microcapillaries. Such erythrocytes are sequestered by the spleen, leading to anemia.

To diagnose HHAs, in addition to family history and the Coombs test, laboratory tests such as the study of osmotic resistance of erythrocytes (RBCOR), the test of eosin-5′-maleimide binding to erythrocytes (EMA test) and the measurement of erythrocyte indices (mean corpuscular hemoglobin concentration (MCHC), mean corpuscular volume (MCV), sphericity index (SphI)) are used.

This study demonstrates for the first time the potential of a method for measuring erythrocyte filterability for diagnosing hereditary spherocytosis. The method is currently in the analytical development stage [34]. The aim of this study was to compare its diagnostic capabilities with other instrumental methods for diagnosing hereditary spherocytosis.

## 2. Results

### 2.1. Comparison of Different Tests Used to Diagnose HHAs

The aim of this study was to compare the erythrocyte filterability test with other diagnostic methods for HS and to evaluate its specificity and sensitivity.

**Figure 2:**
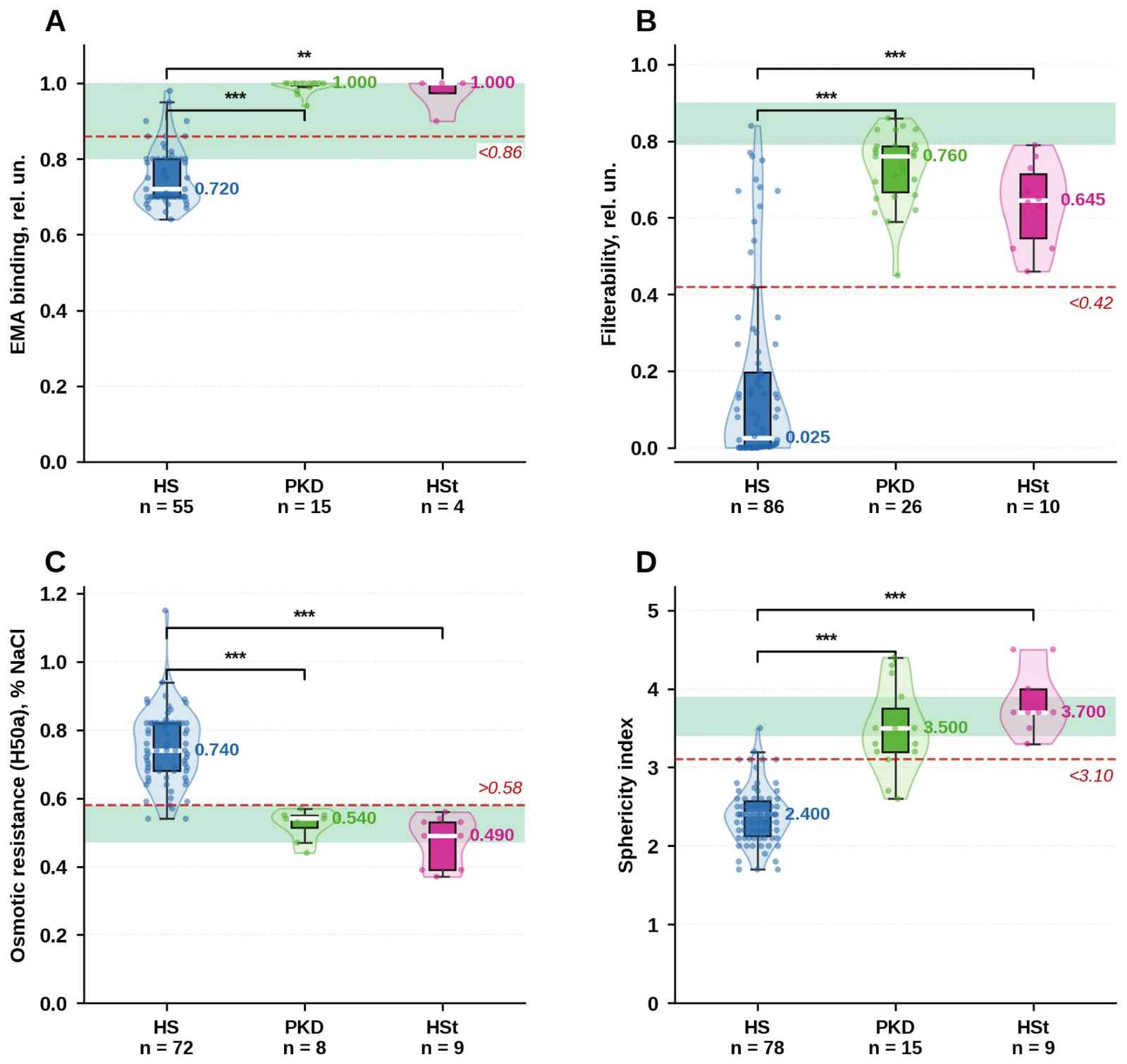
The values of EMA test (**A**), filterability (filter pore diameter 3.5 µm, Ht 1%) (**B**), RBC osmotic resistance (concentration of NaCl (in %) at which 50% of cells lyse after 24 h of incubation at 37 °C) (**C**), sphericity index (**D**) in the groups of the patients with hereditary spherocytosis (HS), pyruvate kinase deficiency (PKD), and hereditary stomatocytosis (HSt). The box sizes represent the 25th to 75th percentile of all measured values, with the white horizontal lines indicating the medians and the whisker range presenting 95% confidence intervals. So-called “violin plots” of the data distribution width are also shown. Red dotted lines present the cut-off values for HS diagnosis. In all the tests, the significance of differences between the patient groups was determined using the Kruskal–Wallis H test and Dunn’s post hoc test with Holm-Bonferroni correction (for 3 pairwise comparisons). The differences were considered significant at *p* < 0.05. The shaded areas represent normal ranges for all tests (ranges including from 5% to 95% of all healthy donor results). ** *p* < 0.01; *** *p* < 0.001.

The values of red blood cell filterability (3.5 μm filters), EMA test, red blood cell osmotic resistance (RBCOR), characterized by the NaCl concentration (in %) causing 50% lysis of red blood cells after 24 h of incubation at 37 °C (H50a), and the sphericity index values obtained in patients with HS, HSt, and PKD are presented in Figure 2. All parameters measured in each patient group are presented in Table S1 in the Supplementary Materials. The calculated medians and 95% confidence intervals of the values for each test in all groups are presented in Table 1.

As shown in our previous work [35], patients with HS typically had low filterability values, but as the number of samples tested increased, it became apparent, that within the HS group, patients can be divided into subgroups with very low (in the range of 0–0.42 relative units) and higher filterability (Figure 2B). In this study, the proportion of patients with higher filterability was 14.0%. The filterability cut-off value for this division (red dotted line) was obtained by calculating the receiver operating characteristic curve (ROC curve) for HS and was 0.42 (see below). None of the other tests demonstrates the emergence of a second subgroup of patients with different result values.

Figure 2 shows that, despite this, the method of measuring RBC filterability reliably distinguishes the HS group from the other groups studied. The reasons for dividing these patients into subgroups remain unclear. Since the entire group of patients with HS is analyzed as a single entity, such separation will undoubtedly reduce the sensitivity of the filterability method for diagnosing HS, calculated using the ROC curve. All methods reliably distinguish HS from HSt or PKD, but none of them reliably distinguishes between HSt and PKD. When using the filterability method, all patient groups differ significantly from the control group. Filterability values for control groups using different filters and hematocrit (Ht) are presented in Figure S1 and Table S2 in the Supplementary Materials.

### 2.2. Filterability Measurement Using Filters with Pore Diameter 3 µm

**Figure 3:**
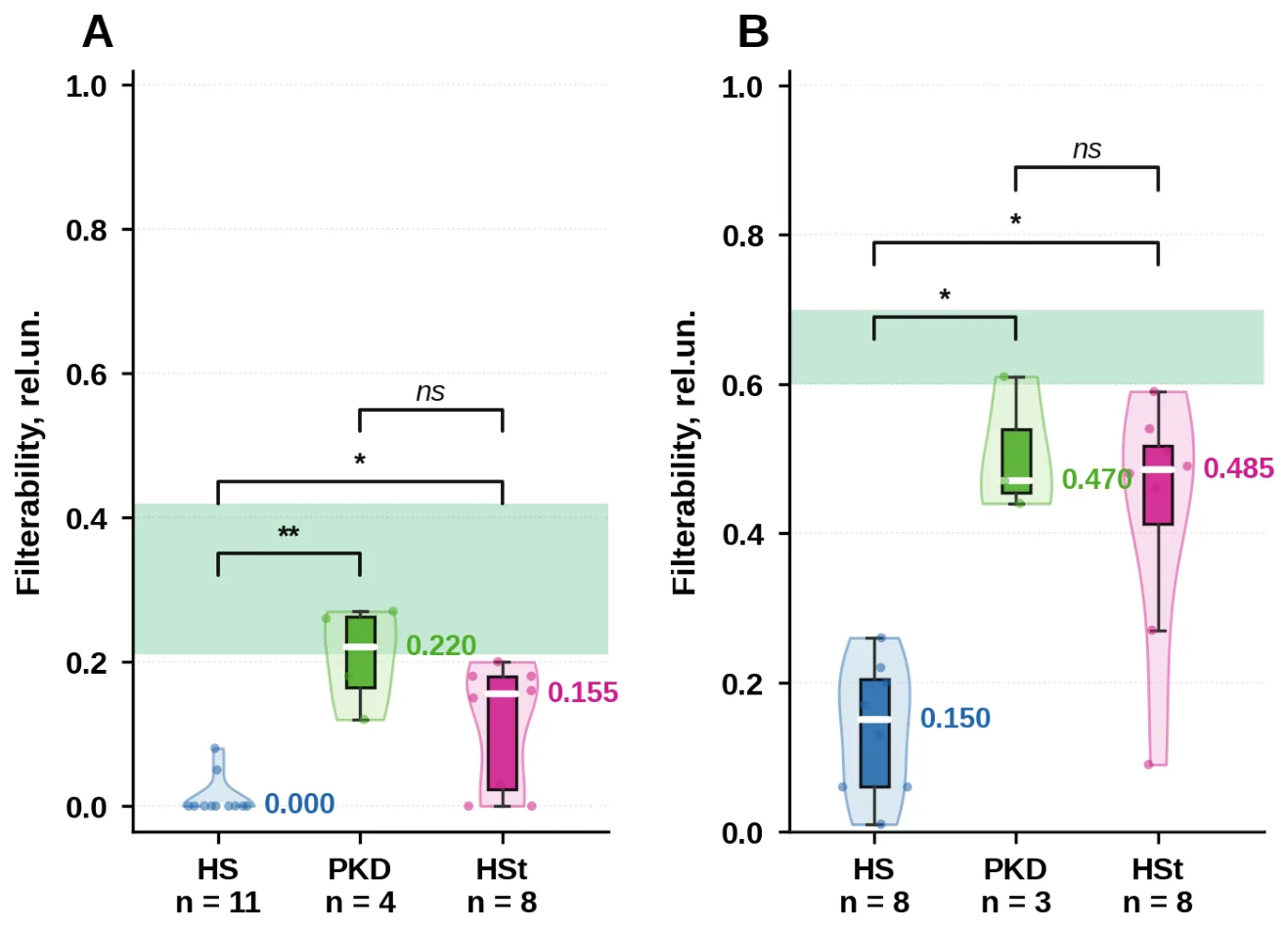
Filterability of erythrocytes from patients with HS, PKD, and HSt measured using filters with a pore diameter of 3.0 μm. (**A**)—Hematocrit of the tested erythrocyte suspension is 1%. (**B**)—Hematocrit of the suspension is 0.1%. The box sizes represent the 25th to 75th percentile of all measured values, with the white horizontal lines indicating the medians and the whisker ranges corresponding to 95% confidence intervals (CI). “Violin plots” of the data distribution width are also shown. Normal ranges (shaded areas) for healthy donors were defined as ranges that included 5% to 95% of all measured values for each experiment type. The number of blood samples from normal donors was *n* = 16 and *n* = 7, for suspensions with a hematocrit of 1% and 0.1%, respectively (see Figure S1 in the Supplementary Materials). In all the tests, the significance of differences between the patient groups was determined using the Kruskal–Wallis H-test and post hoc Dunn’s test with Holm-Bonferroni correction. The differences were considered significant at *p* < 0.05. *ns*—The difference is not significant; * *p* < 0.05; ** *p* < 0.01.

Preliminary results of the study of erythrocyte filterability on filters with a pore diameter of 3 µm were obtained from a small consecutive subgroup of patients admitted to the clinic. The analysis was conducted blinded (staff were unaware of the results of other studies). The results of the molecular genetic analysis were also obtained much later. The results of this study in patients with different HHAs are presented in Figure 3 and in Table S3 in the Supplementary Materials. Some patients were tested in parallel on both filter types. The results showed that all patients with genetically confirmed HS and high filterability on 3.5 µm filters had a significant reduction in filterability compared to the normal value using 3 µm filters. Thus, a reduction in filter pore size (and hematocrit) can help to confirm the diagnosis of HS even in these patients. However, the obtained results cannot yet be considered definitive due to the small sample size of experimental samples for these filters. Further studies are needed to confirm their suitability for detecting HS.

### 2.3. Sensitivity and Specificity of Different Methods for Diagnosing HS

**Figure 4:**
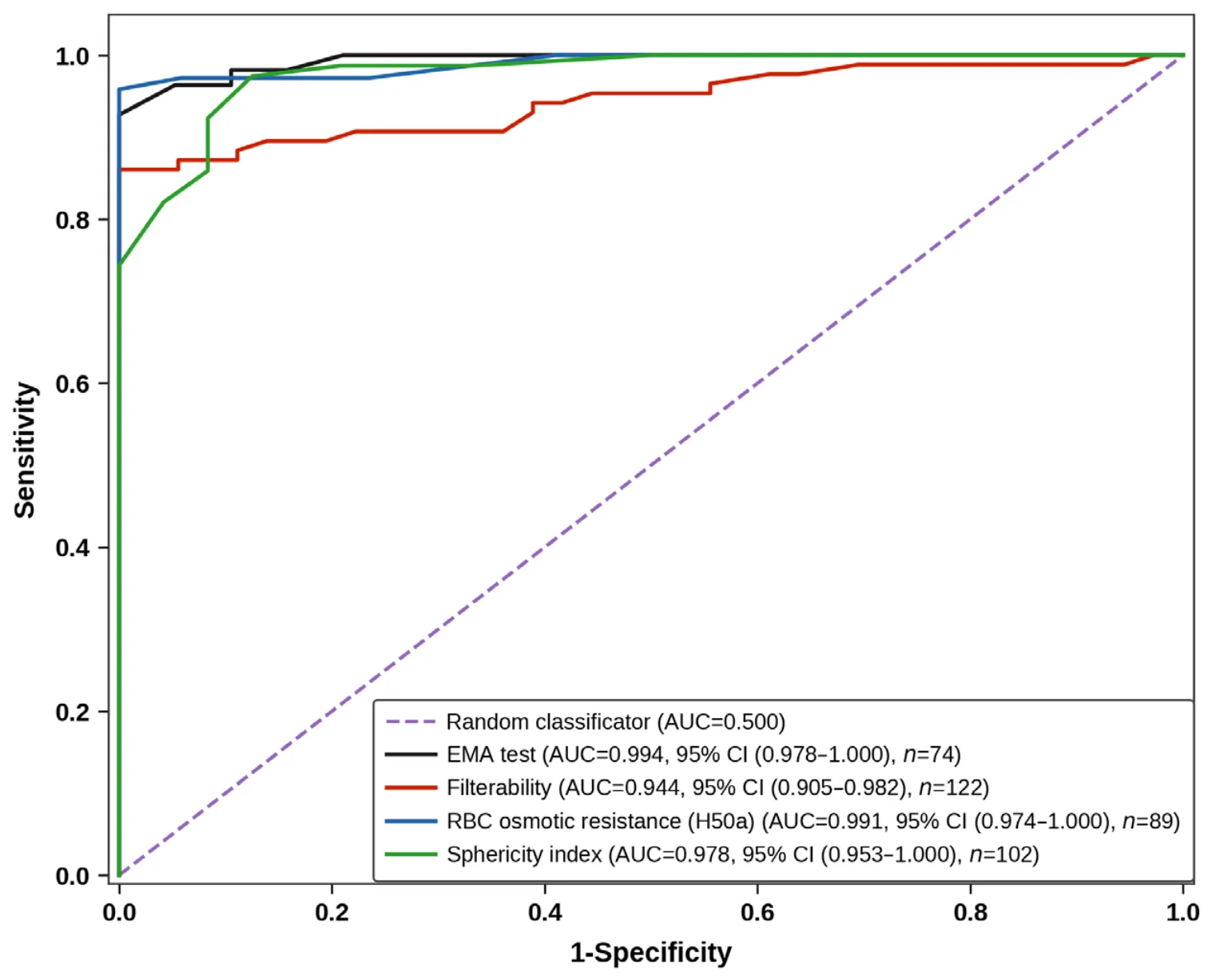
ROC curves constructed for diagnostics of HS using all studied methods. Total number of patients *n* = 126. 95% confidence intervals were calculated using the bootstrap method.

To assess the sensitivity and specificity of different methods in diagnosing HS, the corresponding ROC-curves were constructed for methods of measurement of RBC filterability (at pore diameter of 3.5 µm and Ht 1%), EMA test, RBCOR method, and measuring erythrocyte sphericity index (Figure 4). The values of sensitivity and specificity obtained for each of the studied methods are shown in Table 2.

The sensitivity of various tests for the diagnosis of HS was also studied by pairwise comparison of the different methods using McNemar’s test [36,37]. In this case, when comparing two methods, the total number of pairs tested is compared with the sum of the numbers of false-negative and false-positive results. Because the RBC filterability cut-off determined in this study for HS diagnosis (<0.42 rel. un.) lies well below the lower limit of the normal range, we used this cut-off, rather than the normal-range boundary, to define the disease region in this analysis. For other tests, the following values accepted in the Center were used as the disease region cut-off: EMA test < 0.80 rel. un.; RBCOR > 0.58% NaCl and Sphericity index < 3.40. With such restrictions, the EMA test missed more HS patients than Filterability (but this is not significant, *p* = 0.1892), and significantly more than RBCOR and Sphericity index (*p* = 0.0074 and *p* = 1.526 × 10^−5^, respectively). Filterability missed significantly more than Sphericity index (*p* = 0.0117). Filterability and RBCOR, as well as Sphericity index and RBCOR did not differ significantly from one another (*p* = 0.0654 and *p* = 0.25, respectively). All pairwise comparison data are presented in full in Supplementary Materials (Section S8, Table S8).

### 2.4. Comparison of Areas Under the ROC Curves Obtained in the Diagnosis of HS by Different Methods

The ROC curve for the filterability measurement method had a slightly lower AUC value, but, like most other methods, it had high specificity for diagnosing HS. For a more accurate comparison of AUC values for all methods, the DeLong method was used [38]. The results obtained are presented in Table 3.

The data in Table 3 show that differences in the areas under the ROC curves were significant only for the comparison of the filterability measurement method with RBCOR methods. However, the level of this significance was not very high (*p* < 0.05). It is possible that the significance of the differences in these cases was due to the fairly high power of the analysis (*n* = 87 pairs) and not due to a true clinically significant difference in these areas. This is because with a very large sample size, even extremely small differences can produce a small *p* value, despite the practical (clinical) insignificance of the difference. In addition, as mentioned above, the measured differences in AUCs are influenced by the presence of a second subgroup with higher filterability in the filterability data for patients with spherocytosis.

### 2.5. Comparison of the Sensitivity of Different Methods for the Diagnosis of HS in Patients with Different Mutations of Membrane Proteins

**Figure 5:**
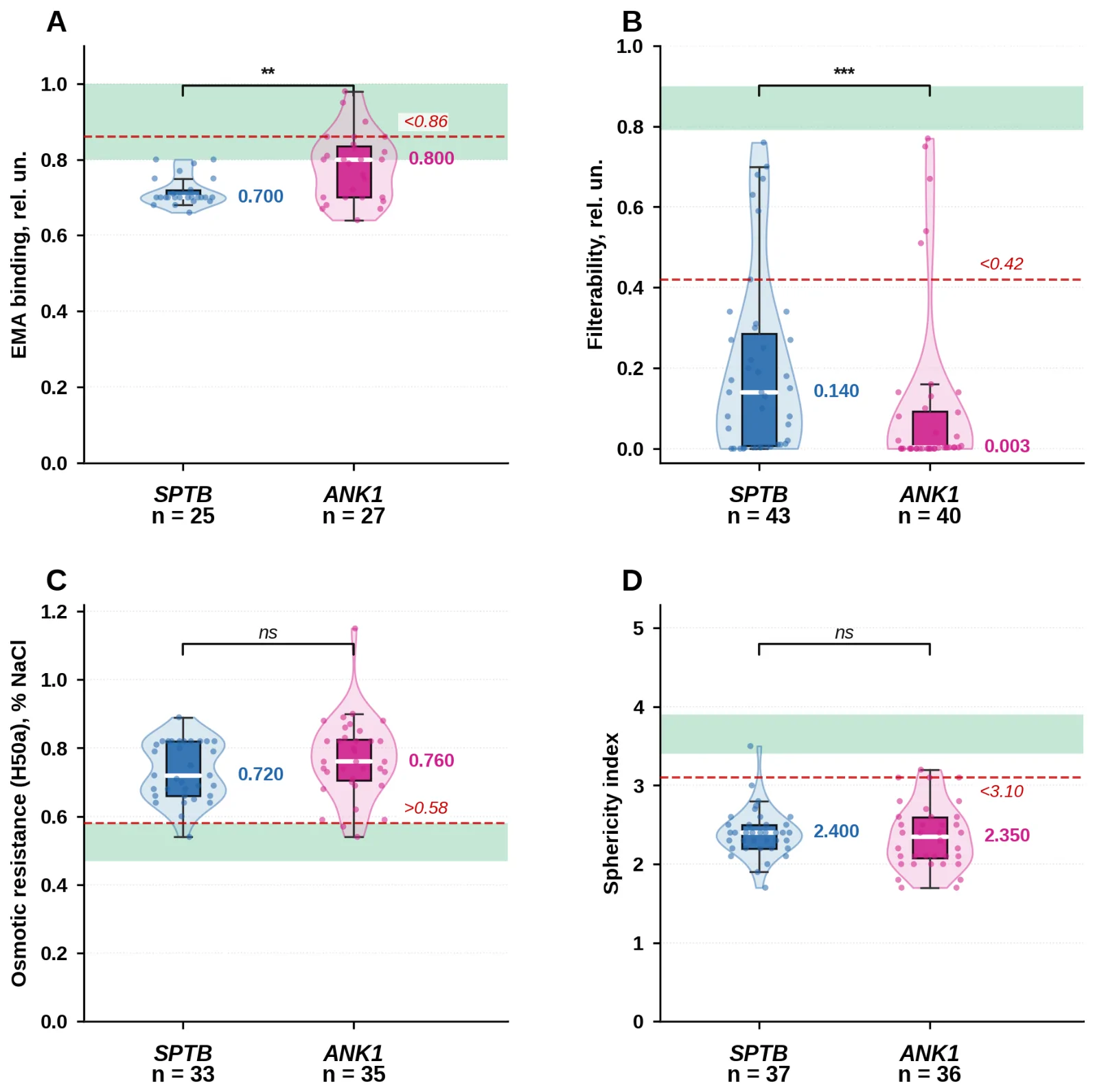
The values of EMA test (**A**), RBC filterability (filter pore diameter 3.5 µm, Ht 1%) (**B**), RBC osmotic resistance (concentration of NaCl (in %) at which 50% of cells lyse after 24 h of incubation at 37 °C) (**C**), and sphericity index (**D**) in the groups of the patients with hereditary spherocytosis, caused by mutations in the *SPTB* or *ANK1* genes. The sizes of the boxes correspond to the 25th and 75th percentiles of all measured values, the white horizontal lines indicate the medians, and the whisker ranges correspond to the 95% confidence intervals. “Violin plots” of the data distribution width are also shown. Red dotted lines present the cut-off values for HS diagnosis. In all the cases, the significance of differences between the patient groups was determined using the Mann–Whitney U test. The differences were considered significant at *p* < 0.05. The shaded areas represent normal ranges including results from 5% to 95% of all healthy donors. The red dotted line represents the cut-off value for each method, determined above when constructing the ROC curves. A diagnosis of HS was considered to be confirmed only if the measured parameter value in the patient fell within the region defined by this cut-off value as the disease region. *ns*—The differences are not significant; ** *p* < 0.01; *** *p* < 0.001.

The composition of the spherocytosis patient population in our study differed significantly from that previously reported in some studies [39,40]. In particular, in our population of patients with HS, there were few patients with mutations in the band 3 protein (gene *SLC4A1*) and α-spectrin (gene *SPTA1*), but almost half were patients with mutations in the gene *ANK1*. Among the 90 patients, 43 had a mutation in the *SPTB* gene (47.8%), 41 had a mutation in the *ANK1* gene (45.6%), and only 6 had other mutations (4 mutations in the *SLC4A1* gene (4.4%) and 2 in the *SPTA1* gene (2.2%)). This allowed us to compare the relative sensitivity of studied methods for detecting spherocytosis caused by mutations in the *SPTB* and *ANK1* genes encoding different erythrocyte membrane proteins. Figure 5 presents the results obtained. Comparisons between paired groups were performed using the Mann–Whitney U test. The results are also presented in Table 4.

All studied tests showed quite high sensitivity to HS. However, only the RBC filterability test and the EMA test reliably differentiated patients with mutations in the *SPTB* or *ANK1* gene. Moreover, the EMA test was found to be superior for the *SPTB* gene mutations (100% of such patients were identified by this test as having HS versus 88.9% of patients with a mutation in the *ANK1* gene). Conversely, the filterability test was superior for the *ANK1* mutation (the disease was confirmed in 87.5% of such patients versus 86.0% in the case of *SPTB* mutation). This means that the RBC filterability test may detect some patients with HS who would not be detected by the EMA test.

### 2.6. Preliminary Analysis of the Possible Relationship Between the Number of Microcytes Detected in Peripheral Blood Smears of Patients with HS and the Value of Erythrocyte Filterability in These Patients

**Figure 6:**
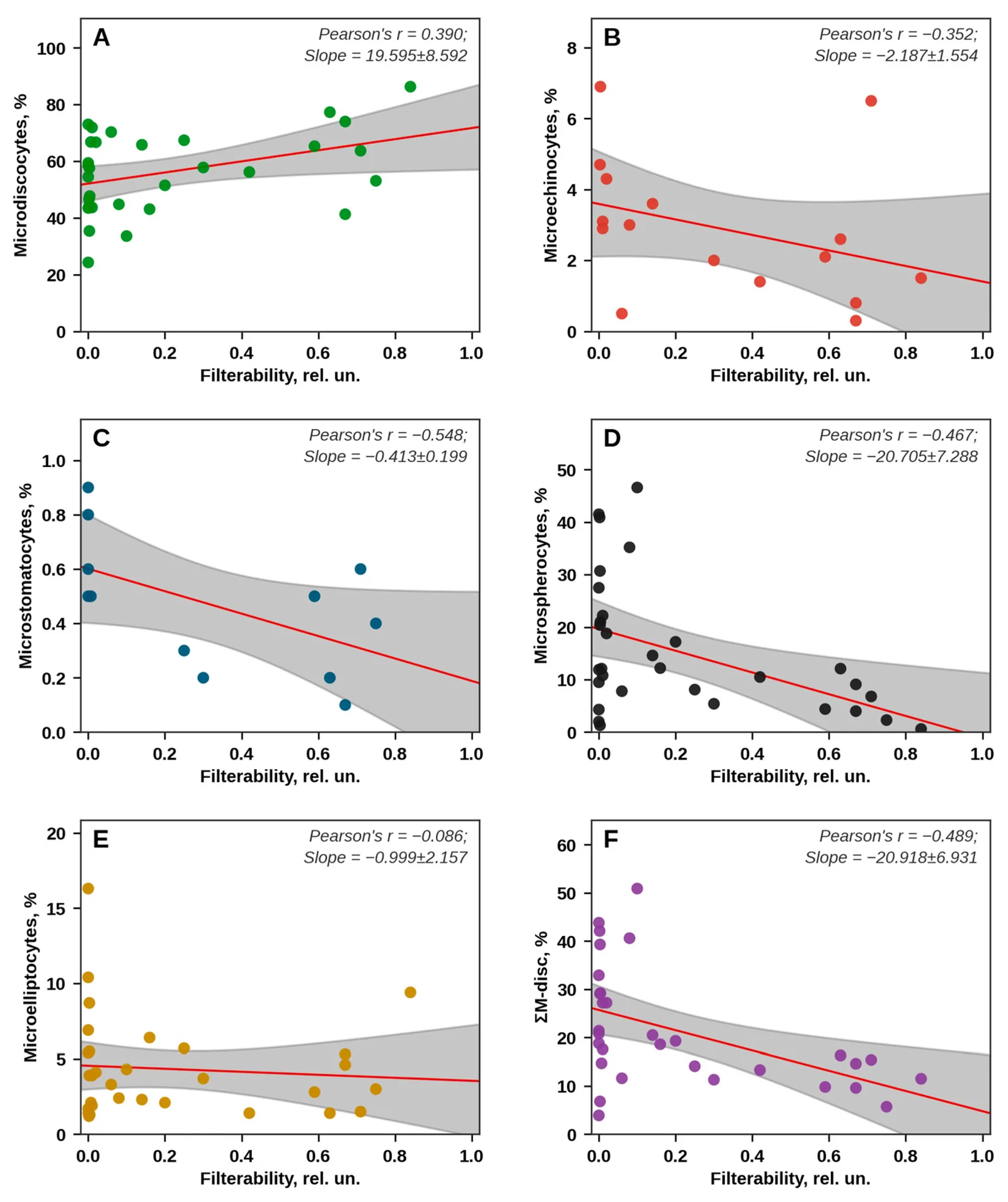
Dependence of erythrocyte filterability of patients with hereditary spherocytosis on the content of microdiscocytes (**A**), microechinocytes (**B**), microstomatocytes (**C**), microspherocytes (**D**) and microelliptocytes (**E**) in the blood, as well as on the sum of all microcytes excluding microdiscocytes (∑M-disc) (**F**), calculated from the data of peripheral blood smears. The red lines represent linear approximations, and the gray shaded areas represent 95% confidence intervals for each dependence. Dots of different colors correspond to the abundance of different microcyte types (**A**–**E**), as well as the sum of all microcytes excluding microdiscocytes (∑M-disc) (**F**). Filterability was measured using filters with pore size of 3.5 µm. Slopes are measured as percentages of corresponding microcytes per 1 filterability rel. unit.

Peripheral blood smear data from 31 patients were analyzed, and the percentage of microdiscocytes, microechinocytes, microstomatocytes, microelliptocytes, and microspherocytes in the blood was calculated (Figure 6). Additionally, the dependence of erythrocyte filterability on the total number of microcytes in the suspension minus microdiscocytes was plotted (Figure 6F).

Different types of microcytes influenced filterability to varying degrees. Thus, an increase in the number of microdiscocytes increased filterability, indicating that these cells have a high deformability, whereas microechinocytes, microstomatocytes, and microspherocytes decreased it. The presence of microelliptocytes had virtually no effect on filterability (see Figure 6 and Table S5 in the Supplementary Materials).

## 3. Discussion

Hereditary spherocytosis is the most common cause of congenital hemolytic anemia. It is diagnosed with a frequency of 1 in 2000–5000 individuals, although a significant number of asymptomatic cases may remain unrecognized [41,42]. The cause of anemia in HS is characterized by a shortened lifespan of RBCs in the bloodstream due to decreased deformability. Qualitative or quantitative deficiencies of membrane proteins caused by genetic mutations disrupt the connection between the inner cytoskeleton of the erythrocyte’s membrane and its outer lipid bilayer. This is accompanied by a decrease in the ability of the RBC to deform, progressive loss of membrane area as RBCs pass through microvessels and narrow slits of venous sinuses in the spleen (a decrease in the cell surface area to volume ratio), and cell sequestration by the spleen, leading to a shortened RBC lifespan and anemia.

Molecular defects leading to HS are highly heterogeneous. These include mutations in genes encoding erythrocyte membrane proteins such as ankyrin, spectrins α and β, band 3 protein, and protein 4.2. Because the ratio of these mutations may vary in different populations, information about these ratios in the literature is contradictory. In work [42], the author writes that the most common causes of HS are band 3 protein deficiency (54%) and spectrin deficiencies (31%); however, in our work, the main proportion of mutations in patients with confirmed HS were mutations in spectrin β and ankyrin, encoded by the *SPTB* (47.8%) and *ANK1* (45.6%) genes.

No single laboratory diagnostic method can detect all cases of HS, so many laboratories use several tests simultaneously. Most often, this is the EMA test and some version of RBCOR measurement [39,43]. The diagnosis is considered established if the patient has a Coombs-negative hemolytic anemia, a reduced EMA test value, reduced osmotic resistance of RBCs, a reduced sphericity index, and an increased mean corpuscular hemoglobin concentration (MCHC). Differential diagnosis of HS is a complex task due to the similarity of clinical and laboratory manifestations in patients with this disease and other forms of HHAs (erythrocyte pyruvate kinase deficiency, dyserythropoietic anemia type II, hereditary stomatocytosis, etc.), as well as because of the low availability of molecular genetic testing [43,44].

Although RBCOR and EMA test are the main methods in the diagnosis of HS, they have limitations. RBCOR is not highly specific for HS, as false negative values can be obtained in 10–20% of HS cases [45,46]. In the work of Y.J. Shim et al. [47], the sensitivity and specificity of RBCOR in the diagnosis of HS were 66% and 81.8%, respectively. However, it has been shown that the RBCOR test using flow cytometry (FCMOF) can increase the sensitivity and specificity to 91.3% and 95.8%, respectively [47]. The RBCOR test also does not differentiate HS from other conditions accompanied by the appearance of spherocytes in the peripheral blood, in particular from autoimmune hemolytic anemia [43,45].

Unlike RBCOR, the EMA test demonstrates higher sensitivity and specificity. The method involves binding the fluorescent dye eosin-5-maleimide (EMA) to the band 3 protein. The N-terminal region of band 3 protein interacts with ankyrin and protein 4.2, which in turn binds to the spectrin cytoskeleton, providing additional stability to the erythrocyte membrane. The absence or decreased expression of any of these erythrocyte membrane proteins leads to disruption of the cell’s cytoskeleton stability and a decrease in the amount of band 3 protein on the erythrocyte membrane surface. As a result, the binding of EMA to the band 3 protein decreases, and, accordingly, the measured fluorescence decreases. The data presented by M. King et al. [43,48] indicate high sensitivity (92.7%) and specificity (99.1%) of the method, which allows the authors to recommend it as the primary method for diagnosing HS. Significant limitations of this method include the need for a flow cytometer, which is not always available in diagnostic laboratories, the high cost of the EMA dye used, and the lack of standard reference values due to the wide range of fluorescence scales on different flow cytometer models.

The sphericity index, erythrocyte osmotic resistance, and the EMA test had (at the cut-off values chosen in this study) high sensitivity in diagnosing HS (96.4%, 95.2%, and 91.8%, respectively). However, the sphericity index had reduced specificity (84.8%). The specificity of erythrocyte osmotic resistance and the EMA test was high (98.6% and 97.2%, respectively). In the case of erythrocyte filterability, specificity was highest (99.1%), but sensitivity was decreased (85.4%).

All methods had high areas under the ROC curve (when compared across all data collected). When comparing these areas with the DeLong method, a significant decrease was observed only for the erythrocyte filterability method. This could be due to both the very large number of samples analyzed when even very minor differences may be formally, but not clinically significant, and the fact that the erythrocyte filterability method distinguishes two subgroups within the group of patients with spherocytosis (Figure 2B).

Apparently, it is this second circumstance that is the main reason for the decrease in AUC for this method. The observed separation of patients in the HS group cannot be explained by poor reproducibility of the method, since a recent study [49] showed that the method has excellent repeatability (when measurements are repeated by the same operator on the same day using the same filter and device, the relative error in repeated measurements is 2.79%) and reproducibility. To study reproducibility, two different groups of donors were studied. The measurements were carried out in different institutions by different operators on different days using different devices [49] (see Figure S2 in Supplementary Materials). These studies showed that, according to Fisher’s test, the relative errors of filterability measurements in different groups are small and do not differ significantly (i.e., the dispersion of the results was homogeneous). A modified Student’s *t*-test [50] showed that the mean filterability values determined in different groups also do not differ significantly, i.e., the test yields unbiased results. When measuring RBC filterability in donor groups (using different filters), as well as in groups of patients with HSt and PKD, no significant scatter in measurement results was observed (Figure 2B and Figure S1 in the Supplementary Materials). All this suggests that the method does, in fact, identify a group of patients who, for some reason, differ from the rest of the population of patients with spherocytosis. The reasons for this distinction are not yet fully understood.

Further studies of these subgroups are needed to determine the exact cause of these differences. The proportion of such patients in our work was 14.0%. We attempted to analyze other available parameters for patients with HS but with different RBC filterability, but so far we have not been able to identify any clear patterns. For most other analyzed parameters, the results were similar to those observed in the general population of patients with HS (Figure 2). Additional data on blood hemoglobin concentrations and total and unconjugated bilirubin levels also did not provide a clear and unambiguous explanation (see Figure S3 and Table S4 in the Supplementary Materials). However, they did reveal a trend that could be interpreted as a more severe condition in patients with poor RBC filterability (they have lower hemoglobin levels but higher levels of both total and unconjugated bilirubin).

The next step in studying different subgroups of patients with spherocytosis was to examine the results of peripheral blood smears. We hypothesized that the presence of all types of microcytes (except microdiscocytes, which, although reduced in size, retain the ability to deform due to their discoid shape) could impair the measured mean RBC filterability. Therefore, we plotted the dependencies of RBC suspension filterability on the amount of each microcyte type (see Figure 6).

It turned out that all the obtained dependencies can be approximated by a linear function (but with a large scatter of results at very low filterability (F < 0.1 rel. un.), where filterability is apparently determined not only by the presence of microcytes, but also by other mechanisms). Moreover, the more microdiscocytes a patient had, the higher the ability of his RBCs to be filtered, which confirms that microdiscocytes have good deformability. Increasing the number of other microcytes reduced filterability to varying degrees. Microspherocytes had the greatest impact on filterability. They accounted for almost 90% of the overall reduction in filterability mediated by the presence of microcytes. Taking all this into account, we obtained a good correlation between the measured value of erythrocyte filterability and the number of microcytes in the patient’s smear when we plotted filterability against the total number of microcytes minus microdiscocytes (Figure 6F). Thus, it can be concluded that in the presence of a higher microdiscocyte content, the overall filterability of the patient’s RBCs is higher. However, what influences the ratio of different microcyte types remains unclear. We believe that HS patients with elevated RBC filterability have some as-yet-unknown mechanisms that improve this filterability. However, the number of such cases is small. Therefore, if HS is suspected, but the patient has a fairly high RBC filterability, a detailed examination is necessary for a more accurate diagnosis.

The lack of an explanation for the occurrence of a subgroup with high filterability in patients with confirmed spherocytosis is a limitation of our study. The characteristics of this subgroup require further investigation. Furthermore, limitations include the small sample size for the PKD and HST patient groups, as well as the lack of data on RBC filterability in other types of hemolytic anemia. Therefore, conclusions from this study about the diagnostic performance of the test should be limited to the selected patient populations. Additionally, the cut-off value used to calculate sensitivity and specificity was derived from ROC analysis of the same dataset used to estimate these parameters; therefore, the reported accuracy estimates may be subject to optimism bias. Validation of this cut-off in an independent patient cohort is warranted. These aspects should be the subject of further research.

## 4. Materials and Methods

### 4.1. Patients and Donors

The study involved 149 consecutive patients admitted to the Dmitry Rogachev National Medical Research Center of Pediatric Hematology, Oncology, and Immunology (Ministry of Healthcare of the Russian Federation, Moscow) who were diagnosed with hereditary hemolytic anemia in accordance with current clinical guidelines. The diagnosis of all HHAs was carried out in accordance with international and Russian clinical guidelines: hereditary spherocytosis—[44,51]; pyruvate kinase deficiency—[52], and hereditary stomatocytosis—[53] (with mandatory molecular genetic confirmation (whole genome sequencing)). All patients or their authorized representatives signed informed consent to participate in the study. No one was excluded from the study. RBC filterability was studied in 126 patients using 3.5 μm filters, and in 23 patients using 3.0 μm filters. To determine normal ranges of RBC filterability, 70 healthy donors were studied (47 using 3.5 μm filters and 23 using 3.0 μm filters) (Table 5). None of the patients had undergone splenectomy or received a transfusion of donor RBCs within the 3 months prior to the study. For all patients, molecular genetic testing results were subsequently obtained (Table S1 in Supplementary Materials).

All patients were divided into groups according to the results of genetic analysis. In the main part of the study, which included 126 patients (in whom RBC filterability was measured on 3.5 μm filters), the HS group included 90 patients, the HSt group included 10 patients, and the PKD group included 26 patients. Basic data of the patients included in the study are presented in Table 5, and the test results obtained for each patient are presented in Table S1 in the Supplementary Materials. There were no siblings among the patients.

The remaining 23 patients were included in testing filters with a 3-μm pore diameter. These data are also presented in Table 5.

### 4.2. Materials

All reagents for buffer preparation were purchased from Sigma-Aldrich (St. Louis, MO, USA). Polyethylene terephthalate membrane filters with cylindrical pores (3.5 μm diameter and 10 μm length, and 3.0 μm diameter and 7 μm length) were provided by the Joint Institute for Nuclear Research (Dubna, Moscow region, Russia).

### 4.3. Standard Tests for Differential Diagnosis of HHAs

Diagnosis of patients was based on family history, the negative Coombs test and standard diagnostic tests for HHAs. These tests included a complete blood count (CBC), determination of red blood cell indices (MCV—mean corpuscular volume, MCHC—mean corpuscular hemoglobin concentration), reticulocyte and normoblast counts, measurement of RBC osmotic resistance, an analysis of EMA (eosin-5′-maleimide) binding to the RBC membrane, erythrocytometry with calculation of the sphericity index, and analysis of a peripheral blood smear. Hemoglobin, total bilirubin, and indirect bilirubin concentrations were also measured. In addition, RBC filterability was measured in patients and donors. All standard laboratory diagnostic tests were carried out in the clinical diagnostic laboratory of the Dmitry Rogachev Center by standard methods with standard sets of reagents used in the hospital [45,46,53,54]. The measurement of erythrocyte filterability was carried out in the Center’s laboratory of Biophysics using the described method [55].

### 4.4. Theoretical Calculation of the Optimal Filter Pore Size for the Best Sensitivity of the Method for Measuring the Filterability of Erythrocytes

The optimal filter pore size for the best sensitivity of the erythrocyte filterability measurement method was calculated using a mathematical model. The calculation results showed that the optimal sizes are:

1.5 µm < pore radius (r) < 1.8 µm (3.0 µm < pore diameter < 3.6 µm);

5 µm < pore length (h) < 10 µm.

The model is presented in more detail in the Supplementary Materials (Section S9, Figure S4).

### 4.5. Preparation of Blood Samples for Filterability Measurement

Blood from all patients and donors was collected in 2.6 mL vacuum tubes (S-monovette, Sarstedt, Nümbrecht, Germany) containing K_3_EDTA as an anticoagulant. To isolate erythrocytes, blood was centrifuged at 1000× *g* for 8 min. The supernatant plasma layer together with the buffy coat was removed. The erythrocytes were washed twice by centrifugation at 1000 *g* for 8 min with a fourfold volume of phosphate-buffered saline (PBS), and then with Tyrode’s solution (135 mM NaCl, 4 mM KCl, 0.33 mM NaH_2_PO_4_, 1 mM MgCl_2_, 11 mM glucose, 2.5 mM CaCl_2_, 10 mM HEPES and 1 mg/mL bovine serum albumin, pH 7.4). Washed erythrocytes were sequentially resuspended in the same solution to a hematocrit of 1% (with an accuracy of 1 ± 0.05%) and used to measure filterability.

### 4.6. Measurement of Erythrocyte Filterability

**Figure 7:**
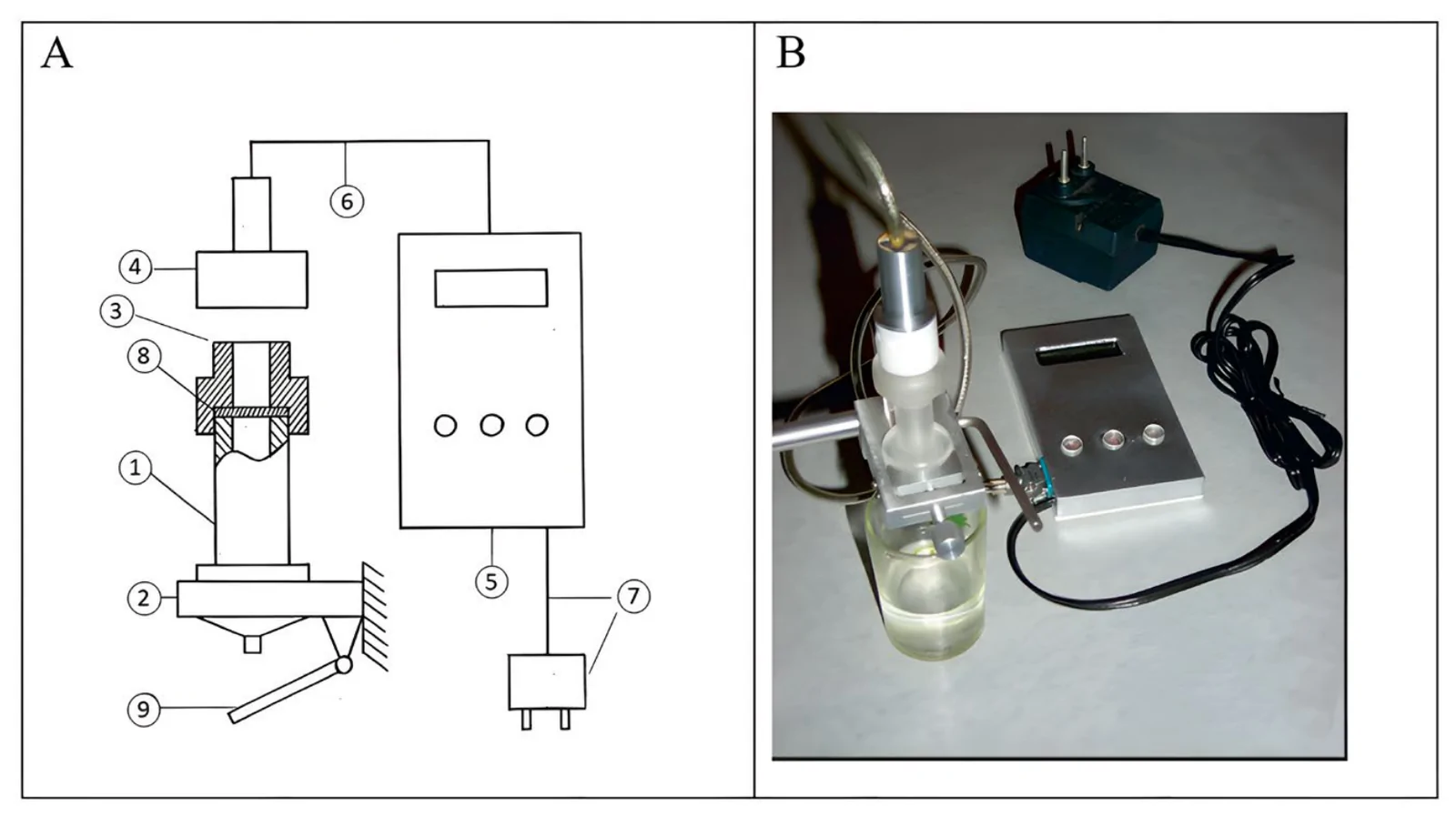
Diagram (**A**) and general view (**B**) of the IDA-01 filterometer. 1—Column; 2—column holder; 3—sample cell; 4—measuring probe; 5—recording unit; 6—3-core PVC cable; 7—2-core PVC cable with adapter for connection to the power supply; 8—polyethylene terephthalate filter; 9—shutter.

The determination of erythrocyte filterability was carried out in accordance with the previously described method [55] using the IDA-01 filterometer patented by us [56,57] (Figure 7). The device was manufactured at the Dmitry Rogachev Center of Pediatric Hematology, Oncology, and Immunology, Moscow, Russia. Using the IDA-01 instrument, the flow time through the same filter was measured, first for 250 µL of buffer (tb), then for 250 µL of a 1% (or 0.1%) suspension of the erythrocytes being studied (ts). Filterability (F) was calculated in relative units as the ratio tb/ts. Each result was obtained by averaging the data of 2–3 parallel measurements.

To minimize the risk of test-review bias, erythrocyte filterability measurements were performed and recorded before the results of molecular genetic testing became available; laboratory personnel conducting the filterability assay were therefore unaware of the patients’ genetic diagnosis and the results of other tests performed at the time of testing [58].

### 4.7. Routine Hematological Indices

Routine hematological indices were measured using XN Series analyzers from Sysmex (Hyogo, Japan).

### 4.8. Genetic Analysis

Genetic analysis was performed using the Hemolytic Anemia panel or using whole-genome high-throughput DNA sequencing (NGS). The enriched DNA library was sequenced on the Illumina NextSeq platform (San Diego, CA, USA).

### 4.9. Determination of Pyruvate Kinase Activity

The activity of pyruvate kinase in the diagnosis of its deficiency was determined by the spectrophotometric method of E. Beutler [59], based on the rate of decrease in the optical density of NADH in the coupled biochemical reactions of pyruvate kinase and lactate dehydrogenase.

### 4.10. Data Analysis

Statistical analyses and data visualization were performed using Python 3.12.3 with the following libraries: SciPy 1.17.1, NumPy 2.4.4, Matplotlib 3.10.8, scikit-learn 1.8.0, and pandas 3.0.2. Each of the biochemical and hematological parameters presented in Table 1 was calculated as the median and 95% confidence intervals (CI) or 1.5 interquartile range (IQR). The significance of the difference between three groups of patients was assessed using the Kruskal–Wallis H-test and post hoc Dunn’s test with Bonferroni correction. The Mann–Whitney U test was used to compare two groups of patients with spherocytosis and mutations in the *SPTB* or *ANK1* genes. The differences were considered significant at *p* < 0.05. Differences in areas under the ROC curves (AUC) were calculated using the DeLong method [38]. Confidence intervals (95% CI) for ROC curves, sensitivity, and specificity values for various HS diagnostic methods were obtained using the bootstrap method with 10,000 iterations. Each iteration used a bootstrap sample (obtained with replacement, equal in size to the original dataset) to determine the optimal Youden threshold and estimate the sensitivity, specificity, and Youden index (J = sensitivity + specificity − 1) in this sample. The same threshold was then applied to the original dataset to obtain the test score, and the difference between the calculated bootstrap values and the test values was recorded as the optimism for that iteration. The final adjusted performance estimate was obtained by subtracting the average optimism over all 10,000 iterations from the apparent value obtained on the full data set (Tables S6 and S7 in Supplementary Materials).

The second method for correcting the obtained sensitivity and specificity values of various methods was a repeated stratified 5-fold cross-validation with 20 independent replications (100 iterations in total). In each iteration, the optimal Youden threshold was determined on the training set, and sensitivity, specificity, and J were estimated on the set-aside test set. The results were averaged across all iterations and replications to obtain a stable cross-validation estimate with standard deviation.

## 5. Conclusions

This study presents an RBC filterability test and demonstrates its potential value for the diagnosis of hereditary spherocytosis. The work corresponds to the analytical development stage of the test and was performed in a cohort that included, in addition to HS patients, individuals with pyruvate kinase deficiency and hereditary stomatocytosis. Because these hereditary hemolytic anemias do not encompass every condition a clinician may encounter in the differential diagnosis of HS, the diagnostic conclusions drawn in this article are strictly limited to the cohort studied.

The test demonstrated high specificity and relatively high sensitivity in diagnosing HS compared to the best instrumental tests currently used for this diagnosis. It is reproducible and not sensitive to variations in the operator and device, which is a necessary condition for a method intended for use across multiple laboratories.

An interesting finding was that, unlike other methods, this test identified a second subgroup of patients with confirmed HS with higher filterability values than in the main group. This is what reduces the test’s sensitivity in the overall patient population. It was shown that this is not due to irreproducibility of the results. However, the reasons for this disparity are not yet fully understood and should be the subject of further research. Patients in this group should be further examined in order to improve diagnostic accuracy.

Further studies are needed to implement the test in clinical practice, but this simple and accessible method for measuring RBC filterability can be readily implemented in any laboratory and represents a reliable alternative to the expensive and not always available EMA test.

## Figures and Tables

**Table 1:** The median values and 95% CI of the tests’ results in different groups of the patients ^(a)^.

Parameter	Group of Patients			Normal Range
	HS	HSt	PKD	
EMA test, rel. un.	0.720 (0.700; 0.790) *n* = 55	1.000 (0.900; 1.000) *n* = 4	1.000 (0.990; 1.000) *n* = 15	0.80–1.00
Filterability (pore diameter 3.5 µm, Ht 1%), rel. un.	0.025 (0.004; 0.100) *n* = 86	0.645 (0.520; 0.730) *n* = 10	0.76 (0.700; 0.782) *n* = 26	0.79–0.90
RBCOR H50a, % NaCl	0.740 (0.715; 0.795) *n* = 72	0.490 (0.390; 0.540) *n* = 9	0.540 (0.470; 0.550) *n* = 8	0.47–0.58
Sphericity index	2.40 (2.30; 2.40) *n* = 78	3.70 (3.50; 4.50) *n* = 9	3.50 (3.20; 3.90) *n* = 15	3.4–3.9

^(a)^ Medians and ranges corresponding to 95% confidence intervals (CI) are presented. The “Normal range” column presents the values accepted at the Dmitry Rogachev National Medical Research Center of Pediatric Hematology, Oncology, and Immunology (these are ranges including from 5 to 95 percentiles of all values in donors). Abbreviations used: HS—hereditary spherocytosis; HSt—hereditary stomatocytosis; PKD—pyruvate kinase deficiency; EMA test—eosin-5′-maleimide binding test to the erythrocyte membrane; RBCOR H50a—RBC osmotic resistance (% NaCl, causing 50% lysis after 24 h of incubation at 37 °C); rel. un.—relative unit. CI were calculated using bootstrap method. Hereditary spherocytosis is diagnosed with EMA test values < 0.80 rel. un., RBCOR > 0.58% NaCl and Sphericity index < 3.40. The erythrocyte filterability region, which was defined in this work as corresponding to the diagnosis of HS, is <0.42 rel. un. (see below in Section 2.3).

**Table 2:** The sensitivity and specificity of various methods for HS diagnostics ^(a)^.

Parameter (Number of Measurements, *n*)	AUC 95% CI (L; U) (Bootstrap)	Cut-Off, Units	Sensitivity, % 95% CI (L; U) (Bootstrap)	Specificity, % 95% CI (L; U) (Bootstrap)	Youden Index Corrected	
					By Bootstrap Method	By Cross—Validation Method ± SD
EMA test (*n* = 74)	0.994 (0.978; 1.000)	0.86 rel. un.	91.8% (82.7; 97.1%)	97.2 (83.2; 100.0)	0.891	0.849 ± 0.123
RBC filterability (*n* = 122)	0.944 (0.901; 0.979)	0.42 rel. un.	85.4 (77.2; 91.8)	99.1 (90.4; 100.0)	0.845	0.835 ± 0.093
RBCOR (H50a) (*n* = 89)	0.991 (0.974; 1.000)	0.58% NaCl	95.2% (88.5; 98.6)	98.6 (81.6; 100.0)	0.938	0.895 ± 0.118
Sphericity index (*n* = 102)	0.978 (0.953; 1.000)	3.10	96.4% (91.1; 99.3)	84.8 (81.6; 100.0)	0.812	0.791 ± 0.140

^(a)^ AUC—area under ROC curve; CI—confidence interval; L and U—lower and upper limits of 95% CI, respectively; RBCOR—osmotic resistance of RBCs; H50a is the NaCl concentration (in %) at which 50% of erythrocytes are lysed after 24 h of incubation at 37 °C; EMA test characterizes binding eosin-5′-maleimide with the RBC membrane. Youden indices (J = sensitivity + specificity − 1) were corrected using bootstrap method and method of cross-validation (with calculation of SD).

**Table 3:** Comparison of areas under the ROC curves obtained in the diagnosis of HS by different methods using the DeLong method ^(a)^.

Compared Methods		AUC1	AUC2	Δ AUC = AUC1 − AUC2	*p* Value	*n* (Pairs)
1	2					
EMA test	Filterability	0.994	0.943	+0.0506	0.0504	*n* = 70 (*ns*)
EMA test	RBCOR (H50a)	0.994	0.984	+0.0106	0.3737	*n* = 58 (*ns*)
EMA test	Sphericity index	0.997	0.977	+0.0199	0.2496	*n* = 63 (*ns*)
Filterability	RBCOR (H50a)	0.923	0.990	−0.0676	0.0153	*n* = 87 (*)
Filterability	Sphericity index	0.932	0.979	−0.0476	0.0801	*n* = 98 (*ns*)
RBCOR (H50a)	Sphericity index	0.992	0.997	−0.0057	0.3857	*n* = 82 (*ns*)

^(a)^ Abbreviations used: AUC—area under the receiver operating characteristic curve (ROC curve); EMA test—test of eosin-5′-maleimide binding to the erythrocyte membrane; RBCOR—erythrocyte osmotic resistance test (H50a—NaCl concentration (in %) at which 50% of the cells were lysed after 24 h of incubation at 37 °C); *ns*—the difference between AUC values is not significant; *—the difference between AUC values is significant (* *p* < 0.05).

**Table 4:** Comparison of the sensitivity of different methods to hereditary spherocytosis caused by mutations in the *SPTB* or *ANK1* gene ^(a)^.

Method	Mutation in Gene	Median (95% CI)	*n* Total (∑*n*)	*n^+^* (Disease)	*n^+^*/∑*n* (%)	*p* Value
EMA test	*SPTB* *ANK1*	0.70 (0.70; 0.71) 0.80 (0.70; 0.82)	25 27	25 24	25/25 (100%) 24/27 (88.9%)	0.00823 (**)
Filterability measurement	*SPTB* *ANK1*	0.140 (0.050; 0.200) 0.003 (0; 0.025)	43 40	37 35	37/43 (86.0%) 35/40 (87.5%)	0.00051 (***)
RBCOR (H50a)	*SPTB* *ANK1*	0.72 (0.68; 0.80) 0.76 (0.73; 0.82)	33 35	32 33	32/33 (97.0%) 33/35 (94.3%)	0.11307
Sphericity index	*SPTB* *ANK1*	2.40 (2.30; 2.40) 2.35 (2.10; 2.50)	37 36	36 35	36/37 (97.3%) 35/36 (97.2%)	0.43080

^(a)^ Abbreviations used: EMA test—test of eosin-5′-maleimide binding to the erythrocyte membrane; RBCOR—erythrocyte osmotic resistance test (H50a—NaCl concentration (in %) at which 50% of the cells were lysed after 24 h of incubation at 37 °C); *n^+^*—the number of the patients in whom HS was confirmed by the test studied. The differences between the groups with mutations in the *SPTB* and *ANK1* genes were compared by the Mann–Whitney U test; ** *p* < 0.01; *** *p* < 0.001.

**Table 5:** The basic characteristics of donors and patients in different subgroups.

Subgroup	*n* Total	Age, Years ^(a)^	Sex (M/F) ^(b)^
Filters with pore diameter 3.5 µm and hematocrit 1%			
HS	90	9 (1; 26)	45/46
HSt	10	11 (1; 20)	8/2
PKD	26	8.5 (1; 26)	8/18
Healthy donors	47	36 (19; 75)	35/12
Filters with pore diameter 3.0 µm (hematocrit 1% and 0.1%)			
HS	11	5 (1; 14)	4/7
HSt	8	12 (3; 15)	0/8
PKD	4	6 (1; 8)	1/3
Healthy donors	23	22 (19; 45)	18/5

^(a)^ Values are presented as medians and ranges (minimum to maximum). ^(b)^ Abbreviations used: M—male, F—female; HS—hereditary spherocytosis; HSt—hereditary stomatocytosis; PKD—pyruvate kinase deficiency.

## Data Availability

The original contributions presented in this study are included in the article/Supplementary Materials. Further inquiries can be directed to the corresponding authors.

## Supplementary Materials

The following supporting information can be downloaded at https://www.mdpi.com/article/10.3390/ijms27177654/s1.
