# Supplementary material for: Differential Diagnosis of Anemia Using the Erythrocyte Filterability Method
Source: Int J Mol Sci. 2026 Aug 26;27(17):7654. doi: 10.3390/ijms27177654 (PMC13566791; doi:10.3390/ijms27177654)
Supplement: Supplementary file 1 [file ijms-27-07654-s001.zip › ijms-4502826-supplementary-final.pdf]

Differential diagnosis of anemia using the erythrocyte filterability method

Elena I. Sinauridze, Elizaveta A. Bovt, Dmitry S. Prudinnik, Ivan A. Dolgikh, Larisa Koleva, Soslan S. Shakhidzhanov, Nikita S. Kushnir, Anna S. Suvorova, Evgeniy S. Protasov, Evgeniya A. Brovkina, Ivan A. Chabin, Svetlana G. Kapranova, Svetlana A. Plyasunova, Nataliya S. Smetanina, Fazoil I. Ataullakhanov

SUPPLEMENTARY MATERIALS

S1. Data of various diagnostic tests for the main cohort of patients

The main cohort included 126 patients with hereditary hemolytic anemia (HHA) (pyruvate kinase deficiency (PKD, *n*=26); hereditary stomatocytosis (HSt, *n*=10) and hereditary spherocytosis (HS, *n*=90)). The diagnosis of all patients was confirmed genetically. In addition to the standard tests commonly used to diagnose HHAs, instrumental tests currently considered the best for diagnosing HS were performed. These were fluorometric measurement of eosin-5'-maleimide binding to the erythrocyte membrane (EMA test), characterization of erythrocyte osmotic resistance (RBCOR) by the H50a parameter, which is the NaCl concentration (in %) at which 50% of the cells are lysed after incubation of the RBCs suspension for 24 hours at 37°C, and measurement of the erythrocyte sphericity index (SphI). A new test was also conducted: measuring the filterability of RBCs through artificial polyethylene terephthalate membranes with cylindrical pores of 3.5 μm diameter and 10 μm pore length. Measurements of the EMA test, RBCOR, and SphI were performed in the clinical diagnostic laboratory of the Dmitry Rogachev Center of Pediatric Hematology, Oncology, and Immunology using standard methods [45,46,53,54]. The filterability of erythrocytes was measured in the Biophysics laboratory of this Center using the IDA-01 device and a specially developed method [55-57]. A range of normal filterability values was defined with erythrocytes from healthy donors (n=47) (see Section S2 in the Supplementary Materials). The method showed good repeatability and reproducibility (relative error 2.79%, coefficient of variation 3.5%) and had no systematic error. The detection limit for pathologically altered cells was 0.003% [49].

All data obtained for the examined patients using instrumental diagnostic methods are presented in Table S1.

Table S1. Parameters of all instrumental tests measured in patients in the main cohort, divided into groups according to molecular genetic analysis data. [(a)]

N	Mutation	Diagnosis	EMA test, rel. un.	Filterability, t_b/t_s, rel. un.	RBCOR (H50a), % NaCl	SphI
		Pyruvate kinase deficiency				
1	*PKLR* CH c.932T>C; c.1456C>T	PKD	-	0,83	-	3,6
2	*PKLR* CH c.932T>C; c.1456C>T	PKD	0,99	0,83	0,57	3,5
3	*PKLR* CH c.1583A>T; c.1436G>A	PKD	1	0,79	-	2,6
4	*PKLR* c.1529G>A hom; *SLC2A1* c.114+2T>C hetero	PKD	-	0,79	-	-
5	*PKLR* CH c.1436G>A; c.487C>T	PKD	0,97	0,78	-	3,2
6	*PKLR* CH c.1291G>A; c.1529G>A	PKD	1	0,78	0,54	3,2
7	*PKLR* CH c.1174G>A; c.1456C>T	PKD	1	0,77	-	-
8	*PKLR* c.1529G>A hom	PKD	-	0,76	-	-
9	*PKLR* CH c.1529G>A; del ex 1	PKD	1	0,76	0,55	-
10	*PKLR* c.1456C>T hetero	PKD	-	0,74	-	3,5
11	*PKLR* CHc.1076G>A; c.1456C>T	PKD	1	0,69	0,54	3,3

Table S1 continued						
N	Mutation	Diagnosis	EMA test, rel. un.	Filterability, t_b/t_s, rel. un.	RBCOR (H50a), % NaCl	SphI
12	*PKLR* int 5 c.695-2A>C hom	PKD	1	0,66	-	2,7
13	*PKLR* CH c.101-1G>A; c.1318G>T	PKD	-	0,66	-	-
14	*PKLR* CH c.1231G>T; c.1456C>T	PKD	1	0,61	0,47	3,5
15	*PKLR* CH c.1529G>A; c.460G>A	PKD	1	0,71	-	3,90
16	*PKLR* c.401T>A hom	PKD	-	0,59	-	-
17	*PKLR* c.1529G>A hom	PKD	1	0,45	0,53	4,3
18	*PKLR* CH c.1079G>A; c.1529G>A	PKD	1	0,62	0,44	4,2
19	*PKLR* c.1594C>T hetero	PKD	-	0,79	-	-
20	*PKLR* c.1529G>A hetero	PKD	-	0,84	-	-
21	*PKLR* c.1529G>A hom	PKD	0,98	0,83	-	4,4
22	*PKLR* c.1529G>A hetero	PKD	1	0,7	-	3,3
23	*PKLR* CH c.994G>A; c.1529G>A	PKD	-	0,65	0,55	3,1
24	*PKLR* CH c.665G>A; c.1456C>T	PKD	-	0,86	-	-
25	*PKLR* CH c.948C>A; c.848T>A	PKD	-	0,73	-	-
26	*PKLR* CH c.1130T>C; c.1456C>	PKD	0,94	0,77	-	-
			Stomatocytosis			
27	*PIEZO1* c.7483_7488 dup hetero	DHSt	1	0,63	0,39	4,5
28	*KCNN4* c.1055G>A hetero	DHSt		0,64	0,37	3,5
29	*PIEZO1* c.7483_7488 dup hetero	DHSt	1	0,52	0,39	4,5
30	*KCNN4* c.940T>C hetero	DHSt	1	0,72	0,53	3,7
31	*PIEZO1* c.7285C>T hetero	DHSt	-	0,65	0,53	3,7
32	*KCNN4* c.611C>G hetero	DHSt	-	0,52	0,54	3,7
33	*RHAG* c.790G>T hetero	OHSt	-	0,76	-	-
34	*PIEZO1* c.1792G>A hetero	DHSt	-	0,79	0,49	3,7
35	*PIEZO1* CH c.2605A>C/c.6593G>A	DHSt	0,9	0,46	0,56	3,3
36	*PIEZO1* c.7483_7488 dup hetero	DHSt	-	0,73	0,49	4
			Spherocytosis			
37	*SPTB* c.4873C>T hetero	HS	0,69	0,004	0,79	2,3
38	*SPTB* c.493C>T hetero	HS	нет	0,005	-	2,74
39	*SPTB* c.1912C>T hetero	HS	0,7	0,004	0,82	2,3
40	*SPTB* c.1912C>T hetero	HS	0,7	0,25	0,81	2,4
41	*SPTB* c.1912C>T hetero	HS	0,7	0,18	-	-
42	*SPTA1* c.2253delA hetero; *SPTA1* c.5911-2A>G hetero	HS	-	0,003	-	2,4
43	*SPTB* c.4267C>T hetero	HS	0,68	0,15	0,82	2,4
44	*SPTB* c.4267C>T hetero	HS	0,70	0,20	0,68	2,5
45	*SPTB* c.4267C>T hetero	HS	0,70	0,08	0,82	2,0
46	*ANK1* c.1453_1454 ins CC hetero	HS	0,7	0,003	0,85	2
47	*SPTB* c.2779C>T hetero	HS	0,66	0,05	0,82	-
48	*ANK1* c.4048G>T hetero	HS	0,76	0,003	0,82	2,4
49	*SPTB* c.5692C>T, hetero	HS	-	0,31	0,82	2,4
50	*SPTB* c.332A>G hetero	HS	0,75	0,17	0,82	2,4
51	*ANK1* c.5097-33G>A hetero	HS	0,67	0,08	0,82	2
52	*SPTB* c.3697A>T hetero	HS	0,71	0	0,82	2,2
53	*SPTB* c. 1064+1G>A hetero	HS	0,68	0,00	0,72	2,1
54	*SPTB* c.3175G>T hetero	HS	-	0,14	0,82	-
55	*SPTB* c.971_980del hetero	HS	0,69	0,003	0,82	2,2
56	*ANK1* c.3555G>T hetero	HS	0,67	0,003	0,57	-
57	*ANK1* c.3157C>T hetero	HS	0,86	0,16	0,74	2,8
58	*ANK1* c.5044C>T hetero	HS	0,86	0,002	0,74	1,8

Table S1 continued

N	Mutation	Diagnosis	EMA test, rel. un.	Filterability, t_b/t_s, rel. un.	RBCOR (H50a), % NaCl	SphI
59	*SPTB* c.1941_1942 dup hetero	HS	-	0,003	-	3,5
60	*SPTB* c. 5598T>A hetero	HS	-	0,68	0,6	2,5
61	*ANK1* c.257C>A hetero	HS	0,83	0,77	-	-
62	*SPTB* c.4603G>T hetero	HS	-	0,002	-	-
63	*SPTB* c.4873C>T hetero	HS	0,7	0,42	0,66	2,8
64	*SPTB* del ex 3-4 hetero	HS	0,7	0,14	0,72	2,3
65	*SPTB* del ex 3-4 hetero	HS	0,7	0,63	0,7	2,5
66	*SPTB* c.5179 del hetero	HS	0,8	0,3	0,66	2,7
67	*SPTB* c.237C>G hetero	HS	-	0,13	-	2,3
68	*ANK1* del intr 24, ex 24-25 het	HS	0,81	0,003	-	2,5
69	*ANK1* c.2401A>T hetero	HS	0,95	0,003	0,54	3,1
70	*SPTB* c.2264 dup T hetero	HS	-	0,012	-	нет
71	ANK1 del ex 18-37 hetero	HS	0,7	0,1	0,86	2
72	*ANK1* c.27+6 T>A hetero	HS	0,86	0,14	0,87	2,8
73	*ANK1* c.3157C>T hetero	HS	0,68	0,003	0,89	-
74	*ANK1* c.5359C>T hetero	HS	0,75	0,003	-	-
75	*SPTB* 5-11 ex del hetero	HS	-	0,003	0,89	1,9
76	*SPTB* c.1774 del hetero	HS	0,75	0,67	0,64	2,2
77	*SPTB* c.2805-1G>A hetero	HS	-	0,1	-	-
78	*SPTB* c.1628G>A hetero	HS	0,7	0,7	-	2,4
79	*SPTB* c.748_754 dup hetero	HS	0,72	0,76	0,54	2,4
80	*ANK1* c.157T>C hetero	HS	0,79	0,51	0,7	-
81	*SPTB* c.5753G>A hetero	HS	-	0,22	-	2,3
82	*SPTB* c.5266C>T hetero	HS	0,79	0,19	0,65	3
83	*SPTB* c.1256 dup hetero	HS	-	0,02	0,8	1,7
84	*SLC4A1* c.1142 del hetero	HS	-	0,84	0,65	2,7
85	*SPTB* c.5346_5348 del hetero	HS	-	0,27	0,69	2,4
86	*ANK1* c.3952_3957 delins GAGAC-GGTTCCAGTCCA hetero	HS	-	0,007	0,79	1,7
87	*ANK1* c.838G>C hetero	HS	-	0,67	0,59	3,2
88	*ANK1* c.810+5G>A hetero	HS	-	0,75	0,68	3,1
89	*SPTB* c.1182+1G>T hetero	HS	0,7	0,01	0,75	2,5
90	*SPTA1* c.4339-99C>T hetero; *SPTA1* c.2659C>T hetero	HS	0,8	0	0,74	2,4
91	*ANK1* c.1602+1G>A hetero	HS	-	0	0,9	1,8
92	*ANK1* c.1602+1G>A hetero	HS	-	0	0,62	2,6
93	*SPTB* c.4093C>T hetero	HS	0,77	0,01	0,64	2,2
94	*ANK1* c.4306C>T hetero	HS	-	0	0,8	2,4
95	*SPTB* c.764-2A>G hetero	HS	-	0,34	0,69	2,6
96	*ANK1* c.1282 dup hetero	HS	-	0	0,82	2,4
97	*ANK1* c.5515 del hetero	HS	-	0	-	2,5
98	*ANK1* c.4306C>T hetero	HS	0,8	0	0,73	2,1
99	*ANK1* c.3630-2A>C hetero	HS	0,8	0	0,74	2,6
100	*SPTB* c.546G>A hetero	HS	0,8	0,06	0,68	2,4
101	*SPTB* c.5753G>A hetero	HS	0,7	0	0,79	2,1
102	*SPTB* c.3793G>T hetero	HS	0,7	0,59	0,71	2,6
103	*ANK1* c.3490 dup hetero	HS	0,7	0	0,76	2,2
104	*ANK1* c.4095 dup hetero	HS	0,8	0	0,82	2
105	*ANK1* c.4462C>T hetero	HS	0,7	0,54	0,69	2,4
106	*SPTB* c.2805-22G>A hetero	HS	-	0,08	0,66	2,6

Table S1 continued						
**N**	**Mutation**	**Diagnosis**	**EMA test, rel. un.**	**Filterability, t_b/t_s, rel. un.**	**RBCOR (H50a), % NaCl**	**SphI**
107	*SPTB* del ex 21-24 hetero	HS	-	0,27	-	2,4
108	*ANK1* c.4153C>T hetero	HS	-	0	0,73	2,5
109	*ANK1* c. 5097-33G>A hetero	HS	-	0,03	0,88	2,7
110	*ANK1* c.3252 del hetero	HS	-	0,13	0,82	2,1
111	*ANK1* c.3257 dup hetero	HS	-	0	1,15	1,7
112	*SPTB* c.5898C>T hetero	HS	-	0,34	0,68	2,2
113	*ANK1* c.3630-2A>C hetero	HS	0,8	0	0,74	2,6
114	ANK1 c.1264C>T hetero	HS	-	0,1	0,69	2,1
115	ANK1 c.327+1G>A hetero	HS	-	0,04	0,83	2,1
116	*ANK1* del int 23 c.2559-2	HS	0,64	0	-	2,1
117	*ANK1* c.4104+543 T>A hetero	HS	0,72	0,09	0,88	2,5
118	*ANK1* c.4125 del G hetero	HS	0,9	0,14	0,76	2,1
119	*ANK1* c.4104+4A>G hetero	HS	0,98	0	0,59	-
121	*ANK1* c.3242 del G hetero	HS	0,69	0,02	0,71	2
124	*ANK1* c.1305G>C hetero	HS	-	0	0,76	2,2
122	*ANK1* c.3623_3624 del CT hetero	HS	0,82	-	-	3,1
123	*ANK1* c.352G>A hetero	HS	0,84	-	-	2,3
124	*SPTB* c.3829C>T hetero	HS	-	0	0,64	2,3
125	*SLC4A1* c.1388G>A hetero	HS	0,9	-	0,94	2,4
126	*SLC4A1* c.2102G>A hetero	HS	0,8	-	0,58	3,1

[a)] Abbreviations used: CH – compound heterozygote; hom – homozygote; hetero – heterozygote. EMA test – test of eosin-5′-maleimide binding with the RBC membrane; t_b – buffer flow time through a filter; t_s -erythrocyte suspension flow time through the same filter; RBCOR – RBC osmotic resistance; H50a – the concentration of NaCl (in %) that causes 50% lysis of erythrocytes after incubation of the suspension for 24 h at 37° C; SphI – sphericity index. PKD – pyruvate kinase deficiency; HS – hereditary spherocytosis; DHSt – dehydrated hereditary stomatocytosis; OHSt – overhydrated hereditary stomatocytosis; rel. un. (relative unit) is the ratio of the measured value in the patient to the control value (in healthy donors).

[b)] In all these experiments, erythrocyte filterability was measured using filters with a pore diameter of 3.5 μm.

S2. Determination of normal ranges of the erythrocyte filterability using various filters and hematocrits of erythrocyte suspension

To determine the filterability using different filters or hematocrit (Ht) of erythrocyte suspensions, it is necessary to determine the normal filterability values under these conditions. For this purpose, filterability was determined in the donor groups under each of the conditions. The normal ranges of values were calculated as the ranges comprising 5% to 95% of all filterability values obtained.

The results obtained for each type of conditions are presented in Figure S1 and Table S2.

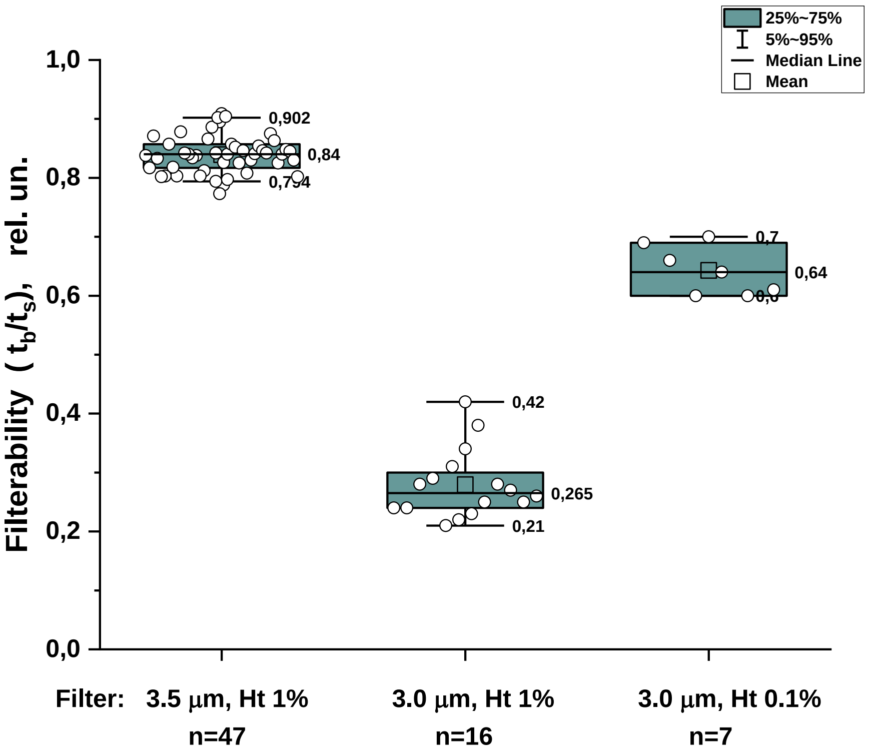

Figure S1. Determination of normal ranges of erythrocyte filterability under various experimental conditions (filter pore diameter and hematocrit of erythrocyte suspension). Box sizes correspond to the 25th and 75th percentiles of all measured values, horizontal lines indicate medians, and the whisker range includes 5%–95% of all measured values in each group.

Table S2. Reference range values for erythrocyte filterability in different experimental conditions determined on RBCs from healthy donors.

Filter (pore diameter and length)	Ht	*n*	Normal filterability range (5%-95% percentiles), relative un.
3.5 µm, 10 µm	1%	47	0.79-0.90
3.0 µm, 7 µm	1%	16	0.21-0.42
3.0 µm, 7 µm	0.1%	7	0.60-0.70

S3. Data on the reproducibility of the results of RBC filterability measurements

The proposed method for measuring erythrocyte filterability has previously been shown to have good repeatability and reproducibility [49]. To investigate the reproducibility of the method, two groups of healthy donors were studied. Filterability measurements were performed by different operators on different days, using different devices, at Hospital No. 40 of the Moscow Department of Health (*n*=15) and in the Biophysics laboratory of the Dmitry Rogachev Center of Pediatric Hematology, Oncology, and Immunology of the Russian Ministry of Healthcare (*n*=31). Each sample was measured in triplicate. The results were averaged. The results were then averaged across all donors, and the mean values for the entire study group and the relative errors for each sample in each group were calculated. Statistical analysis using Fisher's exact test revealed that the relative measurement errors in each group did not differ significantly (the dispersion of the relative errors in each group was homogeneous). Subsequent analysis using a modified Student's t-test revealed that the mean values of donors' RBC filterability determined in each group also did not differ significantly, allowing them to be analyzed together. Thus, the test yields unbiased results (Figure S2).

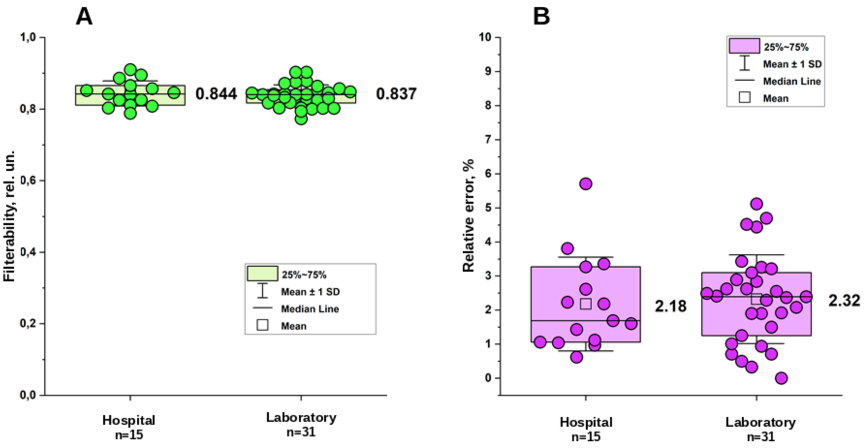

Figure S2. Distribution of donor RBC filterability measurements (A) and relative measurement errors (B) in two series of measurements conducted in different laboratories. Box sizes represent the range from the 25th to the 75th percentile of all measured values. Medians are shown as horizontal lines, the whisker length corresponds to one standard deviation (SD), and the mean values are shown (marked as squares). The figure is adapted from [49] with permission from the Editorial Board of the journal.

Good reproducibility of the analysis results is ensured by measuring the relative flow time of the buffer (250 μl) through the filter to the flow time of an equal volume of RBC suspension through the same filter. This automatically accounts for potential inhomogeneities in the filters used. Furthermore, the analysis is not affected by sample storage time, as the analysis is always performed within 1 hour of blood collection.

S4. Determination of the erythrocyte filterability in patients with different HHAs using 3-μm filters

The bulk of the work was conducted using 3.5-μm pore filters (10-μm pore length) at Ht of 1%. The results of these experiments are presented in Figure 2 and Table 1 of the main text. Small additional donor and patient groups were also recruited to measure RBC filterability using 3.0-μm pore filters (7-μm pore length) and Ht of 1% or 0.1%. Results of these experiments are shown in Figure 3 of the main text and in the Table S3 of Supplementary Materials.

Table S3. Erythrocyte filterability in patients with PKD, HSt and HS, measured using 3.0 μm pore diameter and 7 μm pore length filters and Ht of 1% or 0.1%.[a]

Conditions	Control		PKD		HSt		HS	
	n	**Median (range 5%-95%)**	*n*	**Median (95% CI)**	*n*	**Median (95% CI)**	*n*	**Median (95% CI)**
Ht 1%	16	0.265 (0.21; 0.42)	4	0.22 (0.12; 0.27)	8	0.155 (0; 0.18)	11	0 (0; 0)
Ht 0.1%	7	0.64 (0.60; 0.70)	3	0.47 (0.44; 0.61)	8	0.485 (0.27; 0.54)	8	0.15 (0.06; 0.22)

[a]Abbreviations used: PKD – pyruvate kinase deficiency, HSt – hereditary stomatocytosis; HS – hereditary spherocytosis; 95% CI – 95% confidence interval; Ht – hematocrit of erythrocyte suspension; *n* – number of patients. All confidence intervals were calculated using bootstrap method.

S5. Comparison of hemoglobin levels, as well as total and indirect bilirubin in patients with hereditary spherocytosis, but with different levels of erythrocyte filterability

Hemoglobin, total bilirubin, and unconjugated bilirubin levels were measured in groups of patients with hereditary spherocytosis with low (<0.42) and higher (≥0.42) erythrocyte filterability. The results are presented in Figure S3 and Table S4.

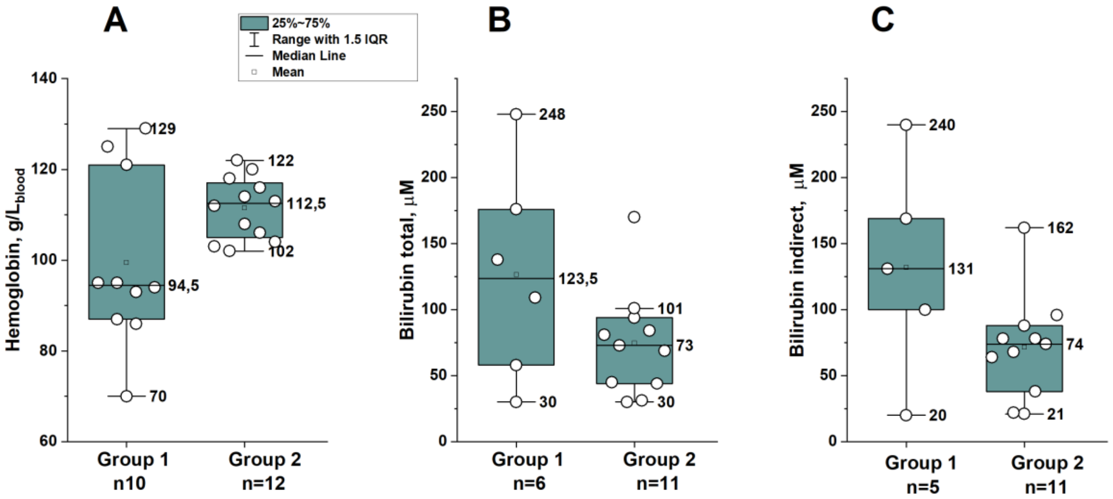

Figure S3. Comparison of hemoglobin (A), total bilirubin (B), and indirect bilirubin (C) levels in two groups of patients with HS: group 1 has a filterability value < 0.42 relative units, group 2 has a filterability value ≥ 0.42 relative units. The box sizes correspond to the 25th and 75th percentiles of all measured values, the horizontal lines indicate the medians, and the range of the whiskers includes 1.5 interquartile ranges**.**

Table S4. Levels of hemoglobin, as well as total and indirect bilirubin in the blood of patients with hereditary spherocytosis with different erythrocyte filterability[(a)].

Group	Hb in blood, g/L$_{blood}$	Bilirubin total, µM	Bilirubin indirect, µM
Group 1 (F<0.42)	94.5 (70; 129); *n*=10	123.5 (30.0; 248.0); *n*=6	131.0 (20.0; 240.0); *n*=5
Group 2 (F≥0.42)	112.5 (102; 122); *n*=12	73.0 (30.0; 101.0); *n*=11	74.0 (21.0; 162.0); *n*=11
p (for groups 1 and 2)	0.106	0.191	0.112

[(a)] Medians and 1.5 interquartile ranges (1.5 IQR) are presented.

We hypothesized that patients in Group 1 with low red blood cell filterability would be in more severe condition. Although the Figure S2 presents some logical differences in the values of all measured parameters in both patient groups, these differences are not significant. This may be due to the small sample size and the fact that these parameters (especially bilirubin levels) exhibit wide variations even within the same group. These variations were greater in the group of patients with low filterability (Group 1).

S6. The influence of different types of microcytes on the filterability of red blood cells in patients with hereditary spherocytosis

Peripheral blood smear data from 31 patients diagnosed with HS were analyzed, and the percentage of microdiscocytes, microechinocytes, microstomatocytes, microelliptocytes, and microspherocytes in the blood was calculated, as well as the percentage of the total microcytes minus microdiscocytes (Figure 6 in the main text). All obtained relationships were approximated by linear functions. The resulting Pearson's approximation coefficients and slopes are presented in Table S5.

Table S5. Pearson coefficients, as well as slope angles for linear approximations of the dependencies of erythrocyte filterability in patients with HS on the content of different types of microcytes in their blood [a].

Type of microcytes	Pearson's coefficient of linear approximation (r)	Slope of linear approximation (% cells per 1 rel. un. of F)
Microdiscocytes	0.390	19.595±8.592
Microechinocytes	-0.352	-2.187±1.554
Microstomatocyte	-0.548	-0.413±0.199
Microspherocytes	-0.467	-20.705±7.288
Microelliptocytes	-0.086	-0.999±2.157
∑M-microdiscocytes	-0.489	-20.918±6.931

(a) ΣM is the sum of microcytes of all types.

S7. Validation of sensitivity and specificity values determined from all available data

To assess whether the sensitivity and specificity values obtained for each diagnostic test were inflated by optimistic bias resulting from using the same dataset for both cut-off value selection and performance assessment, two complementary internal validation approaches were applied.

First, a bootstrap-based optimism correction was performed with 10,000 resamples. In each iteration, a bootstrap sample (obtained with replacement, and equal in size to the original dataset) was used to determine the optimal Youden threshold and estimate the sensitivity, specificity, and Youden index J (J = sensitivity + specificity - 1) in this sample. The same threshold was then applied to the original dataset to obtain the test estimate, and the difference between the bootstrap-corrected value and the calculated test value was recorded as the optimism for that iteration. The final adjusted performance estimate was obtained by subtracting the average optimism across all 10,000 iterations from the value obtained on the full dataset.

A repeated stratified 5-fold cross-validation was then conducted with 20 independent replicates (100 iterations in total). In each iteration, the optimal Youden threshold was determined on the training set, and sensitivity, specificity, and J were estimated on the holdout test set. The results were averaged across all iterations and replicates to obtain a stable cross-validation estimate with a standard deviation. The results obtained are presented in Tables S6 and S7.

The Youden index was used as the primary criterion for threshold selection in all analyses, as it simultaneously maximizes both sensitivity and specificity and represents the point on the ROC curve that maximizes the sum of true positive and true negative results. Confidence intervals for sensitivity and specificity were calculated using the Wilson method. All validation analyses were performed using Python 3.12.3 (SciPy 1.17.1, NumPy 2.4.4, scikit-learn 1.8.0).

The evaluation showed that the adjusted specificity was highest for the RBC filterability method, while the sensitivity was highest for the Sphericity index. The Youden index was highest for the RBCOR method (see Table 2 in the main text).

Table S6. Evaluation of the level of optimism in determining the sensitivity of different methods for diagnosing HS.

Method	Se calculated, %	95% CI, % (Wilson)	Se correct. (bootstrap), %	Se cross-valid. ± SD, %	Optimism |ΔSe|, %	Note
EMA test	92.7	82.7–97.1	91.8	93.5±8.5	0.9	Stable
Filterability	86.0	77.2–91.8	85.4	84.9±7.1	0.6	Stable
RBCOR (H50a)	95.8	88.5–98.6	95.2	94.7±5.9	0.6	Stable
SphI	97.4	91.1–99.3	96.4	92.4±7.1	1.0	Stable

[a] Abbreviations used: Se –sensitivity; Se calculated – value of Se, calculated on all measured date for Se with cut-off calculated on the same data. CI – 95% confidence interval (calculated by Wilson's method). Se correct (bootstrap) – sensitivity value corrected by bootstrap method (10,000 resamples); Se cross-validated - sensitivity value corrected by repeated stratified 5-fold (20 repeats = 100 folds) calculations with averaged standard deviation. |ΔSe| - the level of optimism, i.e. the possible false increase in the sensitivity of the method (in %) due to the fact that the threshold level of the Youden index and the sensitivity itself were determined on the same data sample. EMA test – test of eosin-5′-maleimide binding with the RBC membrane; RBCOR – RBC osmotic resistance; H50a – the concentration of NaCl (in %) that causes 50% lysis of erythrocytes after incubation of the suspension for 24 h at 37° C; SphI – sphericity index.

Table S7. Evaluation of the level of optimism in determining the specificity of different methods for diagnosing HS.

Method	Sp calculated, %	95% CI, % (Wilson)	Sp correct. (bootstrap), %	Sp cross-valid. ± SD, %	Optimism |ΔSp|, %	Note
EMA test	100.0	83.2–100.0	97.2	91.3±13.0	2.8	Stable
Filterability	100.0	90.4–100.0	99.1	98.6±6.1	0.9	Stable
RBCOR (H50a)	100.0	81.6–100.0	98.6	94.8±11.7	1.4	Stable
SphI	87.5	69.0–95.7	84.8	86.7±13.9	2.7	Stable

[a] Abbreviations used: Sp –specificity; Sp calculated – value of Sp, calculated on all measured date for Sp with cut-off calculated on the same data. CI – 95% confidence interval (calculated by Wilson's method). Sp correct (bootstrap) – specificity value corrected by bootstrap method (10,000 resamples); Sp cross-validated - specificity value corrected by repeated stratified 5-fold (20 repeats = 100 folds) calculations with averaged standard deviation. |ΔSp| - the level of optimism, i.e. the possible false increase in the specificity of the method (in %) due to the fact that the threshold level of the Youden index and the specificity itself were determined on the same data sample. EMA test – test of eosin-5′-maleimide binding with the RBC membrane; RBCOR – RBC osmotic resistance; H50a – the concentration of NaCl (in %) that causes 50% lysis of erythrocytes after incubation of the suspension for 24 h at 37° C; SphI – sphericity index.

S8. Paired comparisons of the performance of different tests for the diagnosis of hereditary spherocytosis using McNemar's test

Pairwise comparison of diagnostic sensitivity between methods was performed using McNemar's test [36], applied separately within the disease-positive (HS) and disease-negative (PKD+HSt) subgroups, as recommended for comparing the accuracy of paired diagnostic examinations [37]. Exact binomial McNemar's test was used when the number of discordant pairs was <25; otherwise, the continuity-corrected χ^2 approximation should be applied.

Comparisons were made using a cut-off value of <0.42 for the diagnosis of hereditary spherocytosis in the RBC filterability test. Reference ranges for the remaining tests corresponded to the limits of their normal values. EMA test values <0.80 rel. un.; RBCOR >0.58% NaCl; and Sphericity index <3.40 were taken as disease region boundaries (for HS) (Tables S8A, B). The Youden index was calculated and corrected by bootstrap method in this study.

Table S8. Paired comparisons of the performance of different tests for the diagnosis of hereditary spherocytosis using McNemar's test.

Table S8A. Sensitivity of four diagnostic methods relative to the healthy physiological reference ranges for EMA test, RBCOR and SphI, and at Youden cut-off <0.42 for Filterability of RBCs

Diagnostic Sensitivity in HS Patients - Filterability at Youden Cut-off (<0.42)

PKD and HSt excluded (n=90 HS only). Filterability uses Youden-optimal diagnostic cut-off (boundary value INCLUDED); EMA, RBCOR, Sphericity index use healthy reference range.

Method	Threshold used	n HS tested	Median (95% CI)	Detected (positive)	Missed (negative, FN)	Sensitivity (%)
**EMA test**	< 0.80 (reference norm)	55	0.720 (0.700-0.790)	**36/55**	**19/55**	**65.5%**
**Filterability**	**< 0.42 (Youden cut-off)**	86	0.025 (0.004-0.100)	**74/86**	**12/86**	**86.0%**
**RBCOR (H50a)**	< 0.47 or > 0.58 (reference norm)	72	0.740 (0.715-0.795)	**68/72**	**4/72**	**94.4%**
**Sphericity index**	< 3.40 or > 3.90 (reference norm)	78	2.400 (2.300-2.400)	**77/78**	**1/78**	**98.7%**

Note: Filterability (highlighted yellow) is evaluated at its statistically-derived Youden cut-off (<0.42 rel. un., boundary value counted as positive/abnormal), NOT the healthy reference range (<0.79). This reflects the actual diagnostic threshold used in clinical practice. Other three tests remain evaluated against their healthy physiological reference ranges for comparison.

Table S8B. McNemar's test for pairwise comparison of false-negative rates between diagnostic methods, evaluated in genetically confirmed HS patients with paired measurements available for both methods being compared (based on Table S8A).

McNemar's Test - Pairwise Comparison (Filterability at Youden Cut-off <0.42)

Method 1	Method 2	n paired	Discordant pairs	Method 1 only-missed	Method 2 only-missed	p value & sig
**EMA test**	Filterability	51	21	14	7	0.1892 n.s.
**EMA test**	RBCOR (H50a)	47	15	13	2	**0.0074 ****
**EMA test**	Sphericity index	47	17	17	0	**0.0000 *****
**Filterability**	RBCOR (H50a)	70	11	9	2	0.0654 n.s.
**Filterability**	Sphericity index	74	11	10	1	**0.0117 ***
**RBCOR (H50a)**	Sphericity index	66	3	3	0	0.2500 n.s.

McNemar's exact binomial test (discordant<25) or continuity-corrected chi-square. b = missed-only by Method 1, c = missed-only by Method 2. Only HS patients included. Filterability threshold: Youden cut-off (<0.42, boundary inclusive) for this analysis.

Note: *The RBCOR (H50a) vs Sphericity index comparison had only 3 discordant pairs, below the recommended minimum of 10. The non-significant result for this comparison (p = 0.25) should therefore be interpreted with caution and does not establish equivalence between the two methods.*

S9. Determining the optimal pore size for the best sensitivity in measuring RBC filterability

A mathematical model was developed to determine the pore size for optimal sensitivity of the method. This model assessed the ability of cells to pass through narrow filter pores and was based on the following assumptions:

1. The volume and surface area of the erythrocyte remain constant as it passes through the pore.

2. An erythrocyte is able to pass through a pore if it is able to form the shape shown in Figure S4A (a cylinder with two equal spherical segments at the ends).

It is known that to pass through narrow capillaries, an RBC must have excess surface area (for a given volume) to be able to deform and change shape. The shape shown in Figure S4A has the maximum surface area for a given RBC volume. At the same time, the area of this shape is an estimate of the minimum surface area of the RBC that it can still overcome a pore of radius r and length h.

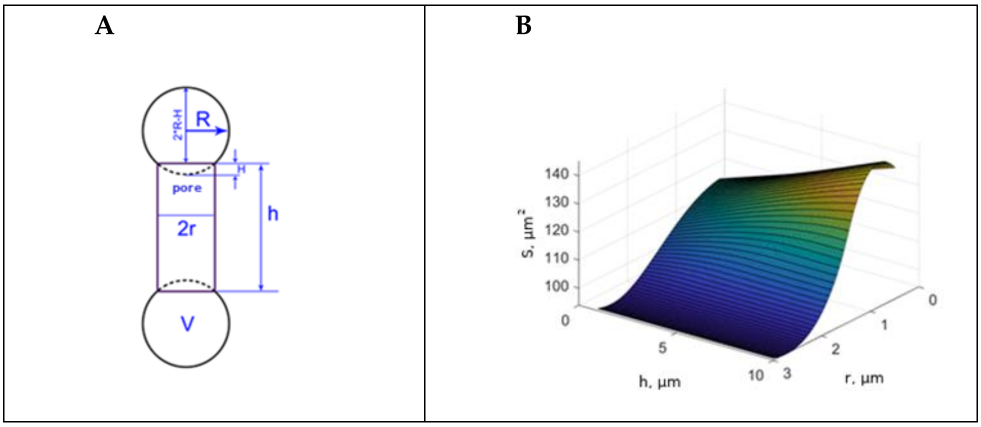

Figure S4. Determining the optimal pore size for the best sensitivity of the method for measuring erythrocyte filterability. A. The location and shape of the erythrocyte in the pore, which allows one to estimate the minimum surface area of the erythrocyte at which it can still pass through the pore with given dimensions. Here, r is the pore radius, R is the radius of the sphere, h is the pore length, H is the height of the spherical segment cut off by the plane of the cylinder (pore) end, and V is the volume of the sphere. B. The dependence of the minimum surface area of the erythrocyte required to pass through the pore on the radius r and the length h of the pore.

The minimum surface area required to pass through a pore was calculated from the system of equations (S1):

$$S = \begin{cases} 2(4\pi R^2 - 2\pi RH) + 2\pi rh, & V \geq \pi r^2 h + \frac{4}{3}\pi r^3 \\ \frac{2V}{r} + \frac{4}{3}\pi r^2, & V < \pi r^2 h + \frac{4}{3}\pi r^3 \end{cases} \quad \text{(S1).}$$

The first equation of the system corresponds to the case shown in Figure S4A, where *R* is the radius and *H* is the height of the spherical segments of the erythrocyte outside the pore, the second corresponds to the limiting case when *R*=*r*, i.e. the segments at the ends are hemispheres of the pore radius.

The unknown height and radius of the segment can be calculated from the system of equations (S2):

$$\begin{cases} H(2R - H) = r^2 \\ \frac{4}{3}\pi R^3 - \pi H^2\left(R - \frac{H}{3}\right) = \frac{1}{2}(V_0 - \pi r^2 h) \end{cases} \quad \text{(S2),}$$

where V_0 is the volume of a red blood cell.

This system was solved numerically in MATLAB with the initial approximations:

$H_0 = R_{max}/2$, $R_0 = (r + R_{max})/2$, where $R_{max} = \sqrt[3]{(3/4\pi\ V_0)}$, is the radius of a sphere with a volume equal to the volume of the RBC.

Substituting the values of H and R obtained from system (1) into system (2) allows us to obtain the smallest surface area of the erythrocyte required to pass through the pore as a function of the pore parameters and the volume of the erythrocyte $S=f(h,r,V_0)$ (Figure S4B).

The resulting dependence was used to determine the optimal parameters (length and radius) of filter pores for diagnosing diseases associated with impaired erythrocyte membrane deformability (spherocytosis, elliptocytosis, etc.). Based on microscopy data from patient erythrocytes and measurements of their filterability, the approximate optimal range of filter parameters was determined: 1.5 μm < r < 1.8 μm, and 5 μm < h < 10 μm.
